# Machine learning algorithm integrates bulk and single-cell transcriptome sequencing to reveal immune-related personalized therapy prediction features for pancreatic cancer

**DOI:** 10.18632/aging.205293

**Published:** 2023-12-12

**Authors:** Longjun Zang, Baoming Zhang, Yanling Zhou, Fusheng Zhang, Xiaodong Tian, Zhongming Tian, Dongjie Chen, Qingwang Miao

**Affiliations:** 1 Department of General Surgery, Taiyuan Central Hospital, Taiyuan 030009, Shanxi, P.R. China; 2 University of Shanghai for Science and Technology, Shanghai 200093, P.R. China; 3 Department of General Surgery, Peking University First Hospital, Beijing 100034, P.R. China; 4 Department of General Surgery, Pancreatic Disease Center, Ruijin Hospital, Shanghai Jiao Tong University School of Medicine, Shanghai 200025, P.R. China; 5 Research Institute of Pancreatic Diseases, Shanghai Key Laboratory of Translational Research for Pancreatic Neoplasms, Shanghai Jiao Tong University School of Medicine, Shanghai 200025, P.R. China; 6 State Key Laboratory of Oncogenes and Related Genes, Institute of Translational Medicine, Shanghai Jiao Tong University, Shanghai 200025, P.R. China

**Keywords:** pancreatic cancer, tumor immune microenvironment, prognostic signature, personalized treatment, single-cell sequencing

## Abstract

Pancreatic cancer (PC) is a digestive malignancy with worse overall survival. Tumor immune environment (TIME) alters the progression and proliferation of various solid tumors. Hence, we aimed to detect the TIME-related classifier to facilitate the personalized treatment of PC. Based on the 1612 immune-related genes (IRGs), we classified patients into Immune_rich and Immune_desert subgroups via consensus clustering. Patients in distinct subtypes exhibited a difference in sensitivity to immune checkpoint blockers (ICB). Next, the immune-related signature (IRS) model was established based on 8 IRGs (SYT12, TNNT1, TRIM46, SMPD3, ANLN, AFF3, CXCL9 and RP1L1) and validated its predictive efficiency in multiple cohorts. RT-qPCR experiments demonstrated the differential expression of 8 IRGs between tumor and normal cell lines. Patients who gained lower IRS score tended to be more sensitive to chemotherapy and immunotherapy, and obtained better overall survival compared to those with higher IRS scores. Moreover, scRNA-seq analysis revealed that fibroblast and ductal cells might affect malignant tumor cells via MIF-(CD74+CD44) and SPP1-CD44 axis. Eventually, we identified eight therapeutic targets and one agent for IRS high patients. Our study screened out the specific regulation pattern of TIME in PC, and shed light on the precise treatment of PC.

## INTRODUCTION

As a malignant tumor of the digestive system, pancreatic cancer (PC) poses a serious challenge to human health with an extremely low five-year survival rate. In the past 30 years, the incidence of pancreatic cancer has steadily increased worldwide [1]. In addition, it is the fourth leading cause of cancer death among men and women of all ages in the United States [2]. Among the traditional treatment modalities including surgical resection and radiotherapy, early surgical resection of pancreatic cancer is considered to be the only possible cure for the malignancy [3]. Noteworthy, only 20% of patients diagnosed with pancreatic cancer can be treated surgically, and even after surgery, most patients will recur and eventually have a seriously poor prognosis. Unfortunately, radiotherapy and chemotherapy for PC also provide limited benefits to patients [4]. Interestingly, advances in immunotherapies, especially immune checkpoint blockade (ICB), have broadened therapy strategies for some historically chemotherapy-refractory malignancies and brought new hope to oncology patients [5]. However, in terms of PC, it has been significantly refractory to ICB therapy. In single-agent ICB and dual-agent ICB studies with anti-PD-1 and anti-cytotoxic t lymphocyte-4 antibodies, overall response rates (ORLs) were 0% and 3%, respectively [6, 7]. These disappointing results (in contrast to the remarkable efficacy of ICBs in other solid tumors) have driven the identification and development of novel immune-related markers in PC that may be key to unlocking immunotherapy as a viable treatment option for pancreatic cancer. Therefore, exploring novel prognostic signatures and drug screening based on the immune level is urgently necessary for delaying the occurrence and development of PC.

Tumor immune microenvironment (TIME) is an indispensable part of tumor progression by providing sufficient nutrients for tumor cell growth and development. The in-depth study of the nature of TIME in the complex evolution of cancer led to a shift from a tumor cell-centered view of cancer development to the concept of a complex tumor ecosystem that supports tumor growth and metastatic spread [8, 9]. The composition of heterogeneous TIME is extremely complex and contains a variety of immunosuppressive cells, including tumor cells, cancer-associated fibroblasts (CAFs), vascular endothelial cells, inhibitory myeloid cells, regulatory T cells (Tregs), and regulatory B cells [10]. These cells and cancer cells can secrete extracellular components, such as extracellular matrix (ECM), matrix metalloproteinase (MMP), growth factors, and transforming growth factor-β (TGFβ), to maintain or disrupt the dynamic equilibrium of the microenvironment and ultimately affect tumor progression [11]. It has been known that these tumor-associated immune cells may possess tumor-antagonizing or tumor-promoting functions. Numerous studies have indicated that the microenvironment plays a vital role in PC progression [12]. Two major features of the pancreatic cancer microenvironment, dense desmoplasia and extensive immunosuppression, facilitated PC cell proliferation and mediated the immune escape via inhibiting the anti-tumor immunity or induction of the proliferation of immunosuppressive cells. Given the temporal heterogeneity, the application of ICB may not be sufficient to maximize the benefit of immunotherapy in PC, and the use of tumor biomarkers involved in maintaining the immunosuppressive microenvironment should also be considered for better outcomes and safety. Hence, it’s necessary for us to explore distinct TIME-related features to guide clinical practice.

In this study, we aimed to explore the immune characteristics of TIME in order to inform the personalized and precise treatment of PC. We identified the immune-related dysregulated genes and constructed the TIME subtype. Additionally, we utilized multiple machine learning algorithms to construct an immune-related signature (IRS) to characterize the relationship between infiltration of immune cells and TIME subtypes and to validate the predictive efficacy of IRS on PC survival outcomes in different cohorts. In fact, we evaluated the sensitivity of chemotherapy and immunotherapy between IRS_high and _low subgroups, and explored the underlying mechanism of how IRS contributes to TIME in PC was also explored based on the results of single-cell sequencing analysis. Eventually, pharmacogenomic datasets are employed to identify potential drug targets and agents and inform immune personalized therapy for pancreatic cancer.

## RESULTS

### Immune-related differential expression genes in pancreatic cancer

**Figure 1:**
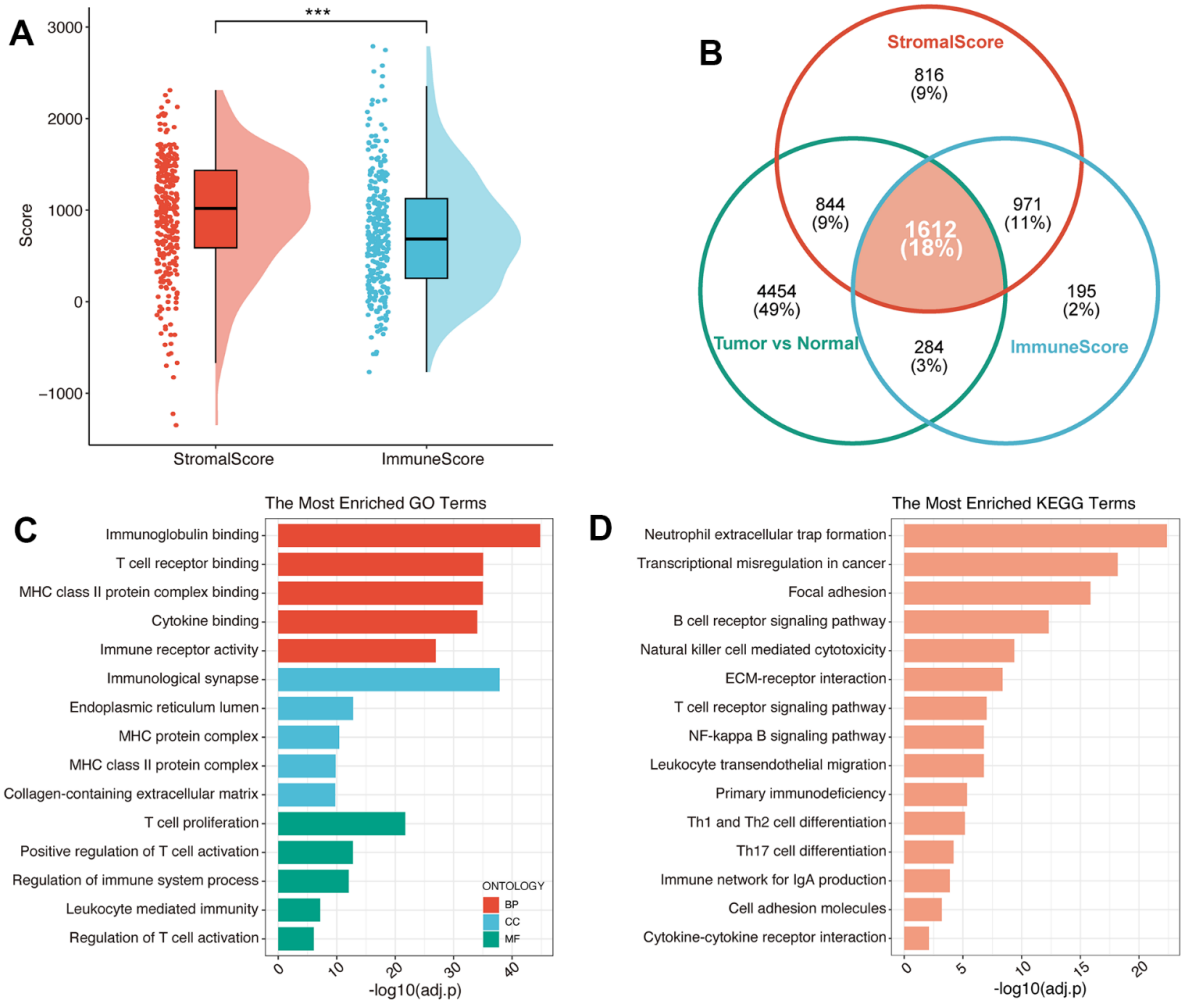
**Identification of immune-related genes in PC.** (**A**) The results of ESTIMATE on the meta-cohort. (**B**) Venn plot exhibited the converged IRGs. GO (**C**) and KEGG (**D**) analysis of IRGs.

PC is known as the “immune desert” due to its unique TIME characteristics. The abundant bone marrow-derived cells and Treg cells in PC can mediate tumor immune escape and cause different levels of immunotherapy resistance through different mechanisms. To further explore the characteristics of TIME in PC, based on meta-cohort, Estimation of STromal and Immune cells in MAlignant Tumours using Expression data (ESTIMATE) algorithm was conducted to calculate the immune and stromal scores of each PC patient (Figure 1A), and the result revealed a high level of infiltrating stromal cells in PC. Subsequently, Differential gene expression analysis in the immune high/low group, stromal high/low group and tumor/normal group suggested (Figure 1B) that 1238 up-regulated and 1824 down-regulated genes were screened compared to the immune low group (Supplementary Table 1). Meanwhile, there were 1919 upregulated and 2324 downregulated genes compared to the stromal-low group (Supplementary Table 2). Additionally, we detected 3731 upregulated and 3463 downregulated genes between pancreatic cancer samples and normal pancreatic samples (Supplementary Table 3). After converging all these three types of differential expression genes (DEGs), 1612 immune-related genes (IRGs) were identified for the following study (Supplementary Table 4). Functional enrichment analysis suggested the function of IRG with potential tumor regulatory mechanisms, and the results suggested IRGs mainly enriched in adaptive immune response and regulation of T cell activation of Gene Ontology (GO) terms (Figure 1C), immune cell receptor signaling and antigen binding pathways of Kyoto Encyclopedia of Genes and Genomes (KEGG) terms (Figure 1D). All these results suggest that PC progression may be related to immune response in the tumor microenvironment to varying degrees.

### Generation of TIME subtype

**Figure 2:**
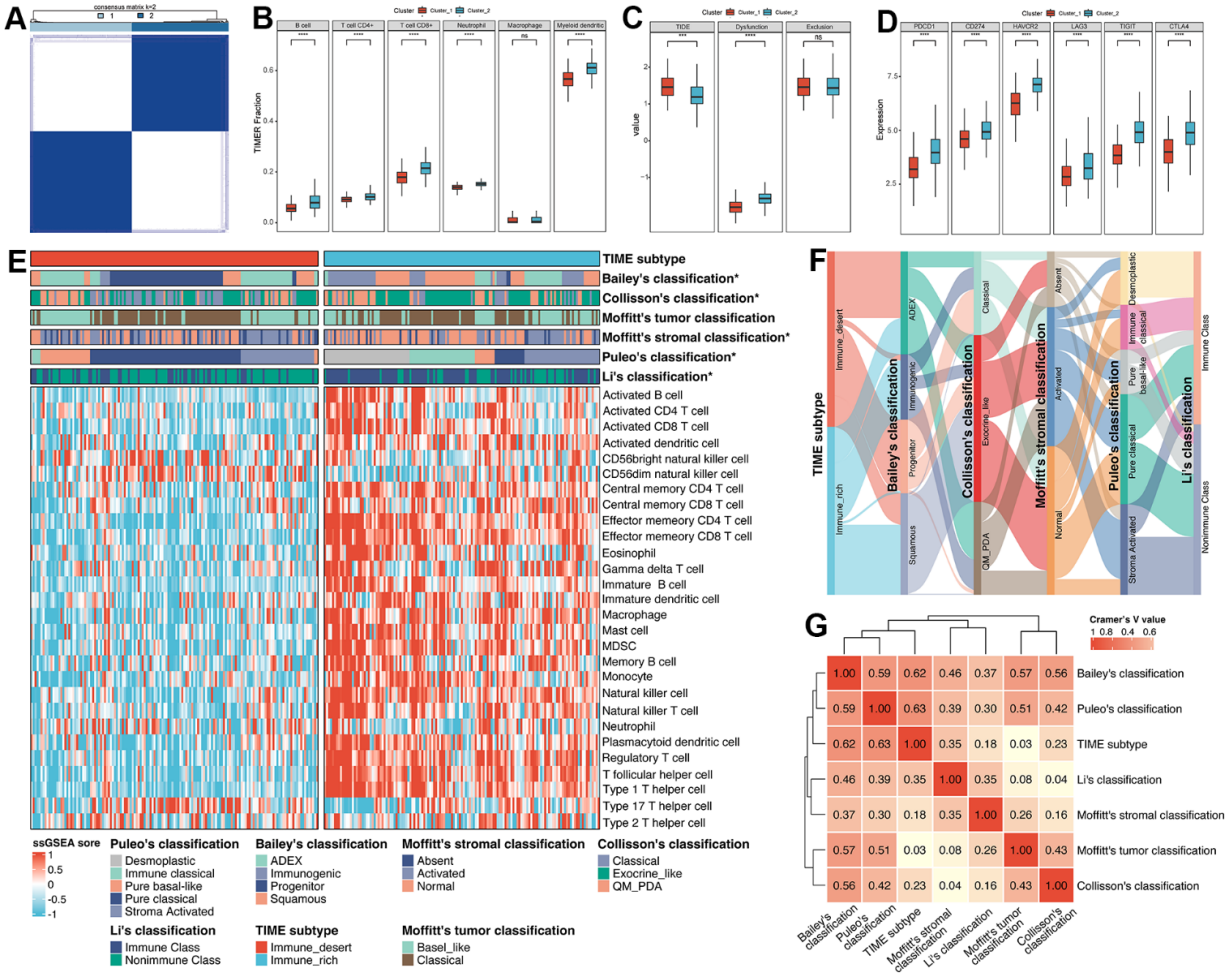
**Exploration of the relationships between the regulation of immune cells and clusters.** (**A**) Unsupervised consensus clustering based on 1612 IRGs. (**B**) The fractions of immune cells between Cluster_1 and Cluster_2. (**C**) The TIDE score between Cluster_1 and Cluster_2. (**D**) The differences in expression of common ICBs among distinct clusters. (**E**) ssGSEA analysis was utilized to estimate the abundance of immune cells. (**F**) The alluvial plot displayed the relationship between the TIME subtype and other molecular classifications. (**G**) Heatmap of Cramer’s V statistic reflected the corrections between seven PC molecular classifications.

Emerging evidence demonstrates that specific expression patterns of TIME could influence the clinical treatment strategies for PC. Hence, we separated PC patients into two clusters (Figure 2A and Supplementary Figure 1A), namely Cluster_1 (n=145, 51.06%) and Cluster_2 (n=139, 48.94%), by an unsupervised consensus clustering algorithm and according to the expression level of 1612 IRGs (optimal cutoff k=2). Interestingly, PCA analysis showed that there are significant differences between these two clusters (Supplementary Figure 1B).

To further identify the correlation between the regulation of immune cells and clusters, Tumor Immune Estimation Resource (TIMER) algorithm was applied to evaluate the abundance of immune cells. As illustrated in Figure 2B, Cluster_2 displayed significantly higher infiltration of immune cells (B cell, CD4+ T cell, CD8+ T cell, neutrophil and myeloid dendritic cell) compared with Cluster_1. Moreover, we performed Tumor Immune Dysfunction and Exclusion (TIDE) algorithm to predict the sensitivity of response to immune checkpoint blockade, including anti-PD1 and anti-CTLA4. Patients in Cluster_2 tend to obtain lower TIDE scores, which means patients in Cluster_2 were sensitive to anti-ICB therapy (Figure 2C). Similarly, we also accessed the diversity in the expression of ICB between Cluster_1 and Cluster_2. Results showed that the expression level of ICB (PDCD1, CD274, HAVCR2, LAG3, TIGIT and GTLA4) in Cluster_2 was obviously upregulated compared to Cluster_1, suggesting that patients in Cluster_2 were more likely to be targeted (Figure 2D). Therefore, regarding the characteristics between those two clusters mentioned above, we manually defined the Cluster_1 as Immune_desert subtype, and Cluster_2 as Immune_rich subtype. ssGSEA analysis also confirmed that the Immune_rich subtype possessed a significant level of innate and adaptive immune cells, including natural killer cells, immature B cells and T cells (all p < 0.0001, Figure 2E). Of note, tumor-suppressing Th1 cells were considerably enriched in the Immune_rich subtype (p = 2.96e-32) compared to tumor-promoting Th2 cells (p = 0.256).

Then, we compared the identified TIME subtype with classical molecular classifications in PC. The marker of Bailey’s classification, Collisson’s classification, Moffitt’s tumor classification, Moffitt’s stromal classification and Li’s classification were utilized to cluster PC patients in the meta-cohort (Supplementary Figure 2A–2E and Supplementary Table 5), and Puleo’s classification was predicted followed the pipeline in Materials and Methods (Supplementary Table 5). Results illustrated that there was no significant difference between Moffitt’s tumor classification and TIME subtype (p = 0.70, Supplementary Table 6), while Bailey’s classification (p < 0.0001), Collisson’s classification (p = 0.0006), Moffitt’s stromal classification (p = 0.0101), Puleo’s classification (p < 0.0001) and Li’s classification (p < 0.0001) obtained significant similarity (Supplementary Table 6). For the comparison of Bailey’s classification, results showed that the proportion of immunogenic subtype was higher and the percentage of progenitor subtypes was lower in Immune_rich subtype versus Immune_desert subtype (35.25% vs 3.45%, 1.44% vs 40.69%, p < 0.0001, Supplementary Table 6). Interestingly, Collisson’s classification, we observed that Immune_rich subtype was composed of a more exocrine-like subtype and a less classical subtype compared to Immune_desert subtype (56.12% vs 41.38%, 14.39% vs 33.79%, p < 0.01, Supplementary Table 6) of Puleo’s classification. For Moffitt’s stromal classification, results demonstrated that Immune_rich subtype possessed a more normal subtype and less activated subtype than Immune_desert subtype (51.80% vs 35.17%, 36.69% vs 44.14%, p < 0.05, Supplementary Table 6). With respect to Puleo’s classification, the frequency of desmoplastic and immune classical was higher within Immune_rich subtype (30.94% vs 0.69%, 23.74% vs 2.76%, p < 0.0001, Supplementary Table 6). On the contrary, we also found a lower frequency of pure basal-like and pure classical subtypes in Immune_rich subtype versus Immune_desert subtype (7.19% vs 18.62%, 10.79% vs 52.41%, p < 0.0001, Supplementary Table 6). In terms of the integration of Li’s classification, we observed that Immune_rich subtype had a positive tendency to enrich in immune class and a negative correlation with nonimmune class, compared to Immune_desert subtype (68.35% vs 33.1%, 31.65% vs 66.90%, p < 0.0001, Supplementary Table 6). Moreover, the correlation between TIME subtype and other published molecular subtypes was quantified by Cramer’s V (Figure 2F, 2G). Results revealed that TIME subtype had the highest correlation with Puleo’s classification (Cramer’s V value = 0.63) and the lowest relationship with Moffitt’s tumor classification (Cramer’s V value = 0.03), probably owing to the deconvolution algorithm applied on tumor cells by Moffitt et al. Additionally, after integrating the TIME subtype and Puleo’s classification, we found that patients with Immune_rich and immune classical subtypes obtained the best survival, while the patients with Immune_desert and pure basal-like subtype had the worst survival (only one patient with Immune_desert and desmoplastic subtype was excluded) (p < 0.0001, Supplementary Figure 3), implying that combination of TIME subtype and Puleo’s classification may guide the prognostic prediction of PC.

### Recognization of key IRGs and construction of IRS for the prognostic prediction of PC

**Figure 3:**
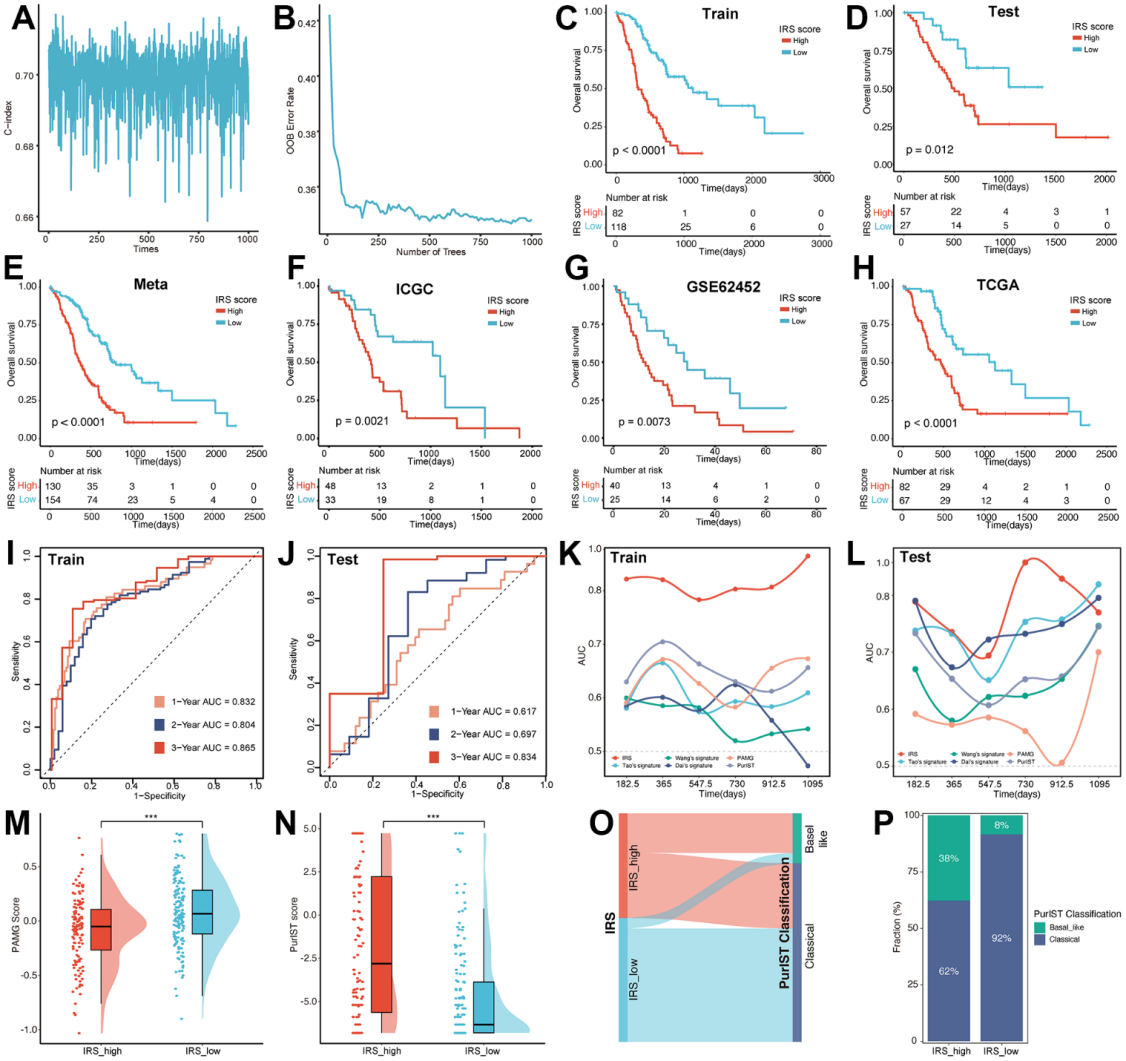
**Construction of the IRS.** The alteration of C index (**A**) and OOB rate (**B**) during 1000-times iteration. The survival analysis was performed based on the training set (**C**), testing set (**D**), meta-cohort (**E**), ICGC (**F**), GEO (**G**) and TCGA (**H**) datasets, respectively. The predictive efficiency of IRS was validated in training set (**I**), testing set (**J**). AUC value was used for the comparison of IRS with other five signatures in the training (**K**) and testing set (**L**). The distribution of PAMG score (**M**) and PurIST score (**N**) between IRS_high and IRS_low group. (**O**) Sankey plot illustrated the distribution of IRS and PurIST classification. (**P**) Distribution of PurIST classification was compared between IRS_high and IRS_low group.

In order to quantize the distinct characteristics among Immune_rich and Immune_desert subtypes, we applied multiple machine-learning algorithms to construct the prognostic signature based on 1612 IRGs. Before proceeding, a filtering procedure was applied to remove genes with low variability and the mean and variance of each gene were standardized to zero and one, respectively. A total of 284 patients in meta-cohort were divided into training set (n=200) and testing set (n=84) at the ratio of 7:3. Robust prognostic IRGs in PC samples were identified using multi-step processes. First, preliminary screening was performed to include 337 prognosis-related IRGs in meta-cohort via univariate Cox regression analysis. Next, bootstrapping method was used to test the genes which passed initial filtering for robustness. We extracted 70% of samples randomly from the training set and performed univariate Cox regression analysis on these samples to assess the correlation between the gene expression and prognosis. This procedure was repeated 1000 times and the 52 IRGs that were incorporated in 90% of resample runs (achieved P < 0.05 in robustness testing) were kept for next step analysis. Then, the random survival forest (RSF) analysis was independently repeated 1000 times, and 8 IRGs with the largest concordance index (C-index) were considered IRS, namely SYT12, TNNT1, TRIM46, SMPD3, ANLN, AFF3, CXCL9 and RP1L1 (Figure 3A, 3B). A risk prediction score model was then developed by these 8 genes using multivariate Cox regression, and the IRS score for each patient was determined by taking the sum of the regression coefficient for each gene multiplied by its corresponding expression value. The IRS score was then normalized from 0 to 1. According to the optimal cutoff value, PC patients were divided into IRS_high and IRS_low subgroups.

To validate the prognostic efficiency of IRS, the survival analysis was performed on the training set, testing set, meta-cohort, International Cancer Genome Consortium (ICGC), Gene Expression Omnibus (GEO) and The Cancer Genome Atlas (TCGA) datasets, respectively. In the 6 internal and external datasets, Kaplan–Meier (KM) curves revealed that the IRS performed well in distinguishing patients with different prognostic statuses (Figure 3C–3H). Also, the univariate Cox analysis showed that SYT12, TNNT1, ANLN, CXCL9 and RP1L1 were risk factors with HR > 1, while TRIM46, SMPD3 and AFF3 were protective factors with HR < 1 (Supplementary Figure 4A), meanwhile, the survival analysis also demonstrated this result (Supplementary Figure 4B–4I). In addition, the receiver operating characteristic (ROC) curve was utilized to verify the prediction ability of the IRS. As shown in Figure 3I, 3J, the IRS model was confirmed effective in predicting the survival of PC patients in 1 year (training set, AUC = 0.832; testing set, AUC = 0.617), 2 years (training set, AUC = 0.804; testing set, AUC = 0.697) and 3 years (training set, AUC = 0.865; testing set, AUC = 0.834).

Previous studies have established several prognostic signatures for PC patients, including Wang’s signature, Tao’s signature, Dai’s signature, pancreatic adenocarcinoma molecular gradient (PAMG) signature and PurIST signature. ROC analysis was performed to confirm whether IRS possessed superior survival prediction ability in PC compared to the five signatures mentioned above. The AUC of the IRS were higher than those of the other three prognostic models in the training set (Figure 3K). Notably, in the testing set, the predictive efficiency was far from satisfactory in 3-year survival, possibly due to the limited number of patients (Figure 3L). To further compare IRS with PAMG and PurIST classification, the PAMG score and PurIST were computed. Results illustrated that the IRS_high subgroup possessed a lower PAMG score and higher PurIST score than the IRS_low subgroup (Figure 3M, 3N). Coincidentally, the Sankey diagram (Figure 3O) and distribution plot (Figure 3P) revealed that the percentage of the Basal-like subtype was significantly lower, and the proportion of Classical subtype was higher within IRS_low subgroup versus IRS_high subgroup (8.44% vs 37.69%, 91.56% vs 62.31%, p < 0.001). The above results fully verified the robustness and predictive effectiveness of our IRS.

### Analysis and validation of differential expression for IRS

**Figure 4:**
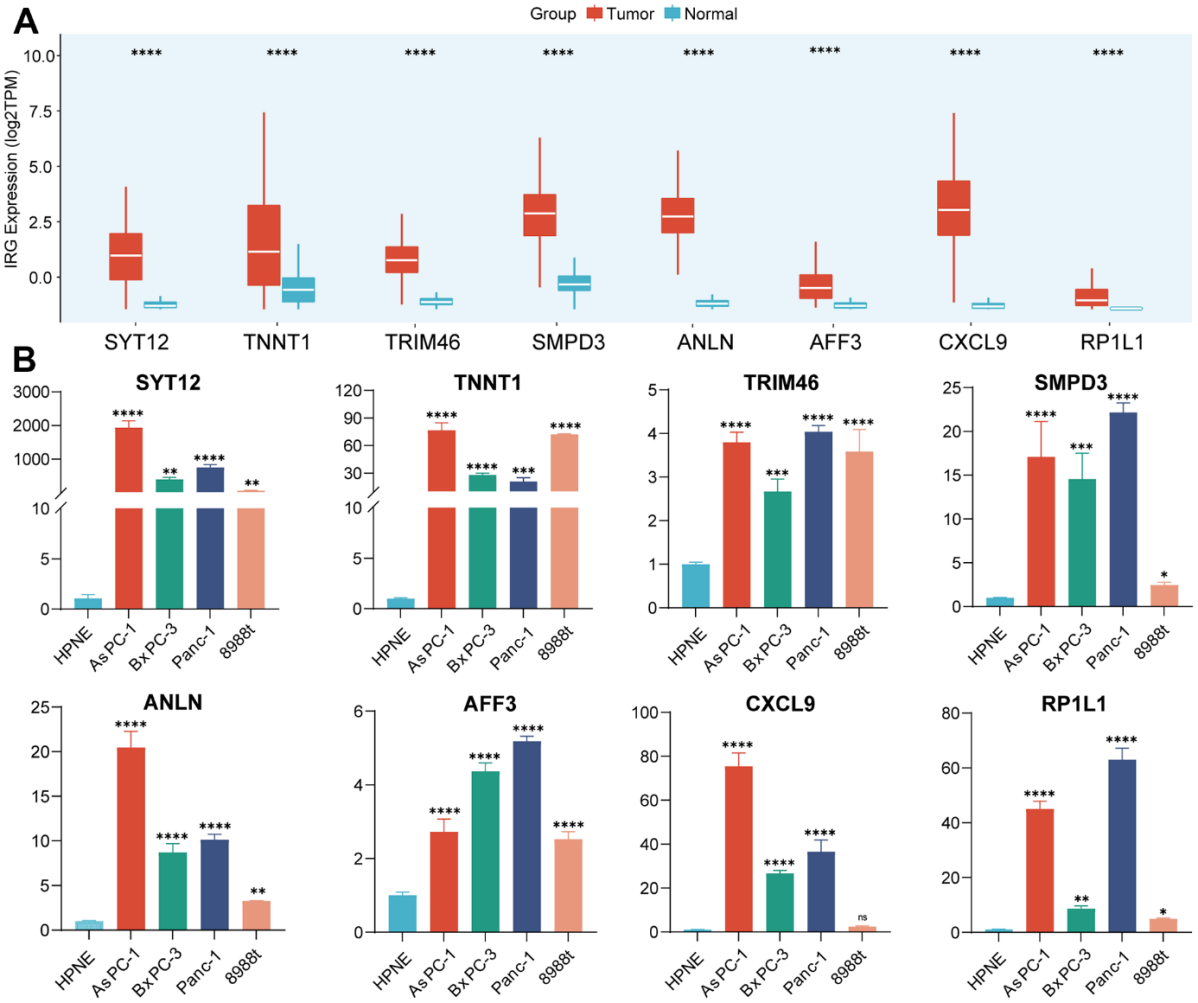
**Validation of the expression of IRGs.** (**A**) The expression of 8 IRGs genes in PC and normal pancreatic tissues. (**B**) RT-qPCR was conducted to validate the expression of 8 IRGs.

As mentioned above, a total of eight genes were selected to construct the IRS based on machine-learning algorithm. Then, we distinguished the aberrant expression of these IRGs in PC and normal pancreatic samples. As illustrated in Figure 4A, all of these IRGs were upregulated in PC samples. qRT-PCR was also performed to validate the differential expression patterns of IRGs between normal pancreatic cell line (hTERT-HPNE) and 4 PC cell lines (AsPC-1, BxPC-3, PANC-1 and PaTu 8988t) (Figure 4B), results suggested that the expression of these eight genes was higher in all four types of pancreatic cancer cells than in normal pancreatic cells. Owing to the significant upregulation of these hub genes, they may serve as potential targets of PC which suggested further research.

### Exploration of IRS-based chemotherapy prediction and potential immunotherapeutic response

**Figure 5:**
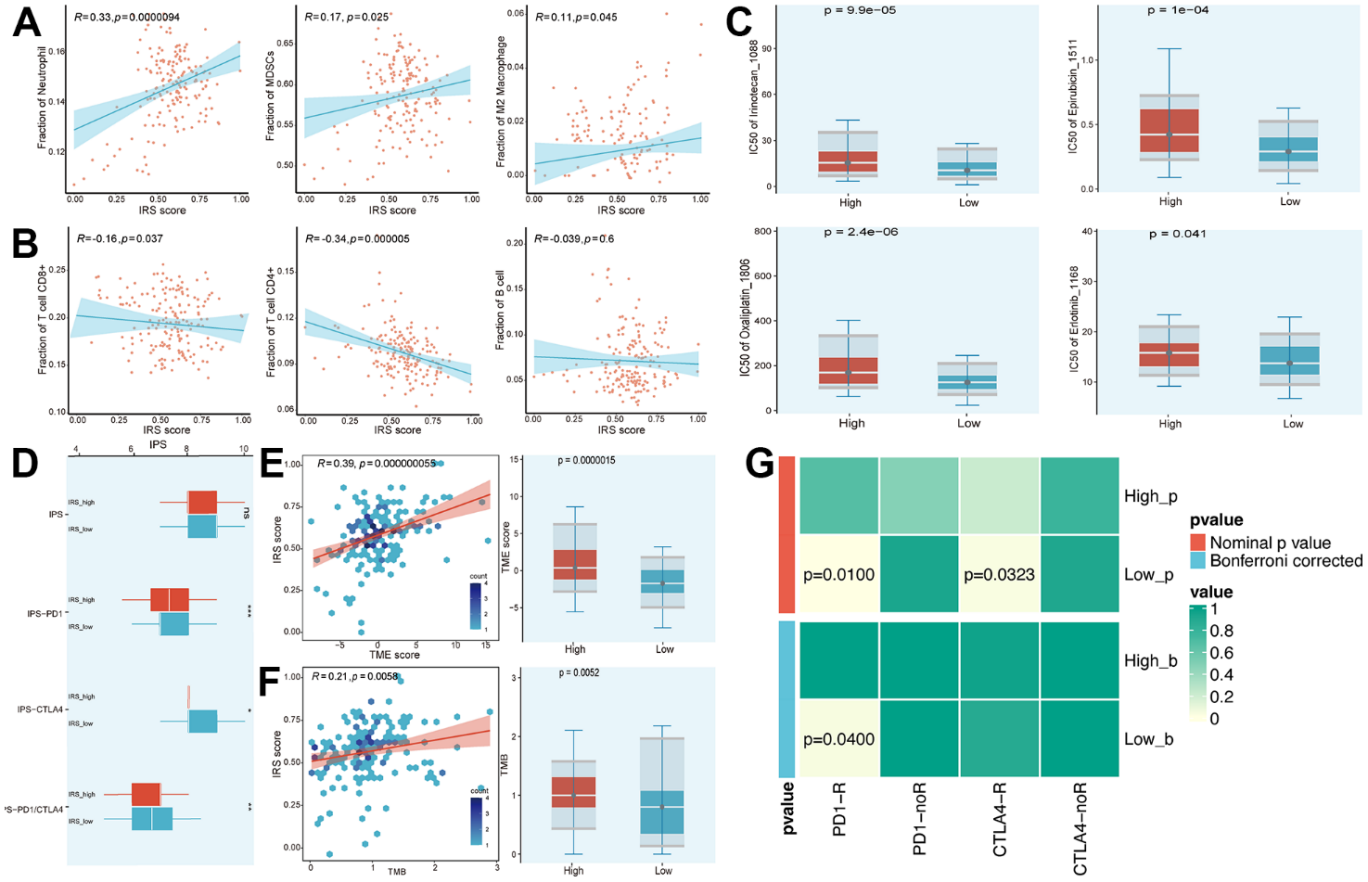
**Prediction of chemotherapy and immunotherapy.** (**A**, **B**) The relationship between the IRS score and the infiltration of immune cells. (**C**) Estimated IC50 of common chemical compounds between IRS_high and _low groups. (**D**) IPS scores between IRS_high and _low groups. (**E**, **F**) The correlation between IRS score and TME/TMB score. (**G**) Contingency table between immunotherapy responses (anti-PD-1 and anti-CTLA-4) and IRS groups based on SubMap analysis.

As the IRS was established based on prognostic IRGs, we first analyzed the relationship between the IRS score and the infiltration of immune cells. The IRS score was positively correlated with neutrophils, myeloid-derived suppressor cells (MDSCs) and M2 macrophages. On the contrary, CD8+ T cells and CD4+ T cells displayed a negative relationship with the IRS score (Figure 5A, 5B). Moreover, we suggested that the IRS may predict the sensitivity of chemotherapy by comparing the IC50 of multiple chemical compounds between IRS_high and _low groups. As shown in Figure 5C, patients who gained a lower IRS score tended to be more sensitive to chemotherapy. In terms of the prediction value of IRS in the treatment of ICB, we calculated the immunophenoscore (IPS), IPS-CTLA4, IPS-PD1 and IPS-PD1-CTLA4 scores, which are quantitative indexes to access the treatment of ICBs, were higher in the IRS_high group (Figure 5D). Furthermore, we compared the distribution of Tumor microenvironment (TME) and tumor mutation burden (TMB) scores in IRS_high and _low subgroups to evaluate whether the IRS could predict the clinical response to ICB therapy. Results exhibited that the TME and TMB scores were higher in the IRS_high subgroup and both had a positive correlation with the IRS score (Figure 5E, 5F). Since the IRS had a remarkable correlation with the TIME of PC mentioned above, we further determined whether the IRS could predict immunotherapeutic response in PC via SubMap analysis. We evaluated the similarity of the expression module of immune-related gene expression profiles between our cohorts and a cohort of 32 melanoma patients receiving ICB therapy [13]. Results illustrated the similarity between patients in the IRS_low group and patients who responded to anti-PD-1 and anti-CTLA4 immunotherapy (Figure 5G). All these results implied that the IRS_low group may have better feedback in immunotherapy compared to IRS_high group, which needs to be further validated in immunotherapy cohorts of PC.

### Single-cell sequencing reveals potential mechanism of TIME regulation by IRS

**Figure 6:**
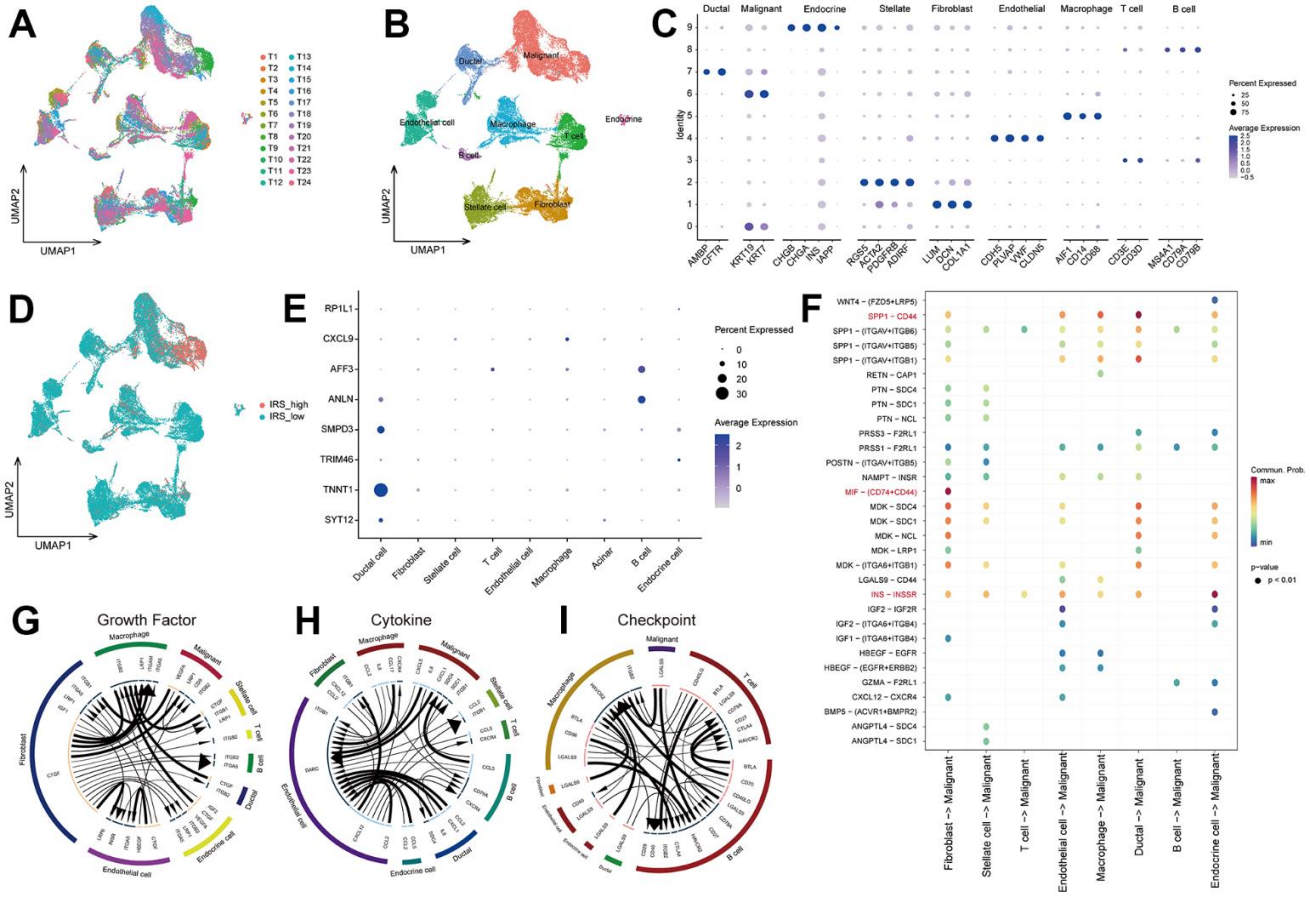
**The underlying mechanism of IRS via scRNA-seq analsysis.** (**A**) The distribution of cells after quality check. (**B**) UMAP plot showing the cells were clustered into 9 types. (**C**) The expression of marker genes in 9 types of cells. (**D**) The distribution of IRS score among 9 types of cells. (**E**) The expression of 8 IRGs in 9 types of cells. Cell-cell communication analysis were implemented via “CellChat” (**F**) and “iTALK” (**G**–**I**) algorithms.

To further recognize the TIME personalized features of pancreatic cancer, scRNA-seq data from 24 PC samples were utilized to reveal the potential mechanism of IRS-promoted PC progression. 22910 cells were screened after quality checks according to the aforementioned research methods. According to the marker genes extracted from the literature, 9 clusters were determined and then annotated to 9 cell types (Figure 6A–6C): malignant, fibroblast, stellate cell, T cell, endothelial cell, macrophage, ductal, B cell and endocrine cell. To unveil the mechanism of the IRS, we evaluated the distribution of IRS scores in 9 cell types. As illustrated in Figure 6D, higher IRS scores were mainly congregated in malignant cells, which could explain the poor prognosis of high IRS PCs. We also checked the expression of IRS in 9 cell clusters. SMPD3 and TNNT1 were significantly expressed by malignant cells, while AFF3 and ANLN were mainly expressed by B cells (Figure 6E). Therefore, we suggested that malignant cells may contribute to the specific TIME in IRS. Cell-cell communication demonstrated that fibroblast had a significant influence on malignant cells via MIF-(CD74+/CD44) interactions, and ductal cells affected malignant cells through SPP1-CD44 interactions (Figure 6F). Meyer-Siegler KL [14] found that blocking MIF-CD74 interactions may provide new targeted specific therapies for androgen-independent prostate cancer. SPP1-CD44 axis was reported to promote the interplay between CAF and enrichment of stemness population in PC [15]. These results demonstrated that fibroblast cells and ductal cells might promote the progression of cancer via MIF-CD74 and SPP1-CD44 axis, respectively. In addition, CTGF-LRP1 interactions between fibroblast cells and malignant cells could also cause the development of cancer (Figure 6G, 6H). In fact, the expression of HAVCR2 and ITGB2 was higher in macrophage and B cells, while the expression of LGALS9 was upregulated in fibroblast cells and malignant cells (Figure 6I). These results revealed that fibroblast cells might prohibit the activation of immune cells, leading to the “immune dessert” status of PC. Hence, IRS may promote the progression of tumor and suppress the immune system in TIME.

### Identification of IRS-related biological processes and drug targets

**Figure 7:**
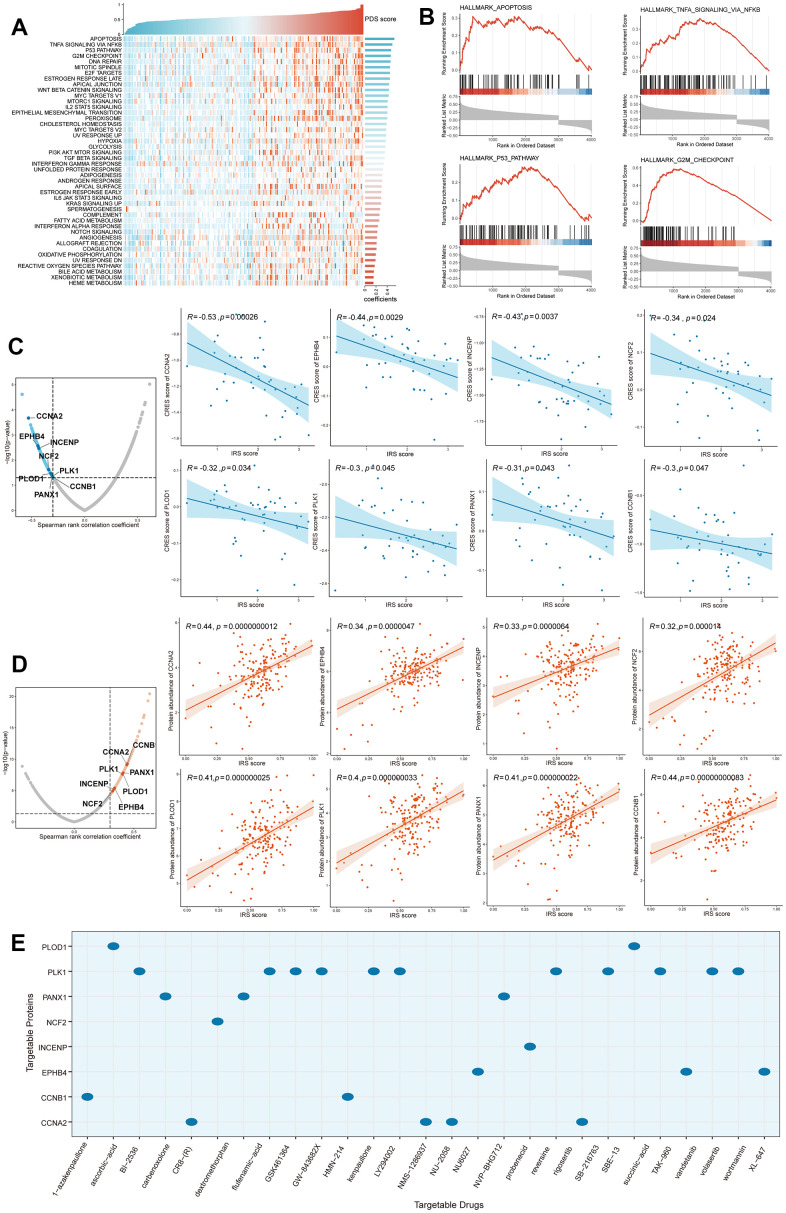
**Identification of IRS-related biological processes and drug targets.** (**A**) Pathifier analysis showing the IRS-related biological processes. (**B**) Top 4 pathways were validated in GSEA analysis. Potential drug targets were screened out form CTRP (**C**) and PRISM (**D**) datasets. (**E**) The corresponding relationship between drug targets and potential targeted drugs.

In order to investigate which biological process plays a critical role in poor prognostic of PC patients who gained high IRS scores, Pathifier and Gene set enrichment analysis (GSEA) analyses were performed to elucidate the potential mechanisms involved in the regulation of PC progression by IRS. Based on gene expression data from both pancreatic cancer and normal pancreatic samples, pathway deregulation score (PDS) was computed via “Pathifier” R package. The correlation between PDS scores and IRS scores helps to evaluate whether a pathway (biological process) may be responsible for the poor prognosis of patients with high IRS scores. “Apoptosis”, “TNFA signaling via NFKB”, “G2M checkpoint” and “DNA repair” pathways ranked top, which means these three pathways may contribute to the malignant phenotype in patients with high PPS scores (Figure 7A). Next, we performed GSEA analysis to validate the above conclusion. Enrichment score of each gene set was calculated and adjusted P-value less than 0.05 was considered significantly enriched. As expected, genes with positive correlation coefficients were also enriched in those four pathways (Figure 7B). Taken together, the dysregulation of apoptosis and cell cycle-related process might play a vital role in the poor prognosis of high IRS patients.

In high IRS patients, Genes significantly positively correlated with IRS may be potential targets for pancreatic cancer precision therapy. To identify targetable proteins (genes) with potential therapeutic implications in high IRS score PC patients, we conducted Spearman correlation analysis between the protein abundance of targetable genes and PPS. A protein with a correlation coefficient more than 0.3 (with P < 0.05) was considered as a poor prognosis-related drug target. Next, we calculated the IRS score for each PC cell line from the Cancer Cell Line Encyclopedia (CCLE) project, and performed the correlation analysis between the CERES score and PPS score based on these cell lines. A lower CERES score of a gene indicates a higher likelihood that this gene is dependent on a given cancer cell line (CCL). Therefore, we considered a gene with a correlation coefficient less than -0.3 (with P < 0.05) as a poor prognosis-dependent drug target. Potential therapeutic drug targets in high IRS score PCs were then considered as targets identified by both analyses above. Finally, 8 potential targets (CCNA2, EPHB4, INCENP, NCF2, PLOD1, PLK1, PANX1 and CCNB1) were screened out (Figure 7C, 7D) and the correlated target drugs were also identified (Figure 7E), which meant that targeting these genes may facilitate the treatment of high IRS PCs.

### Identification of potential agents for high IRS score PCs

**Figure 8:**
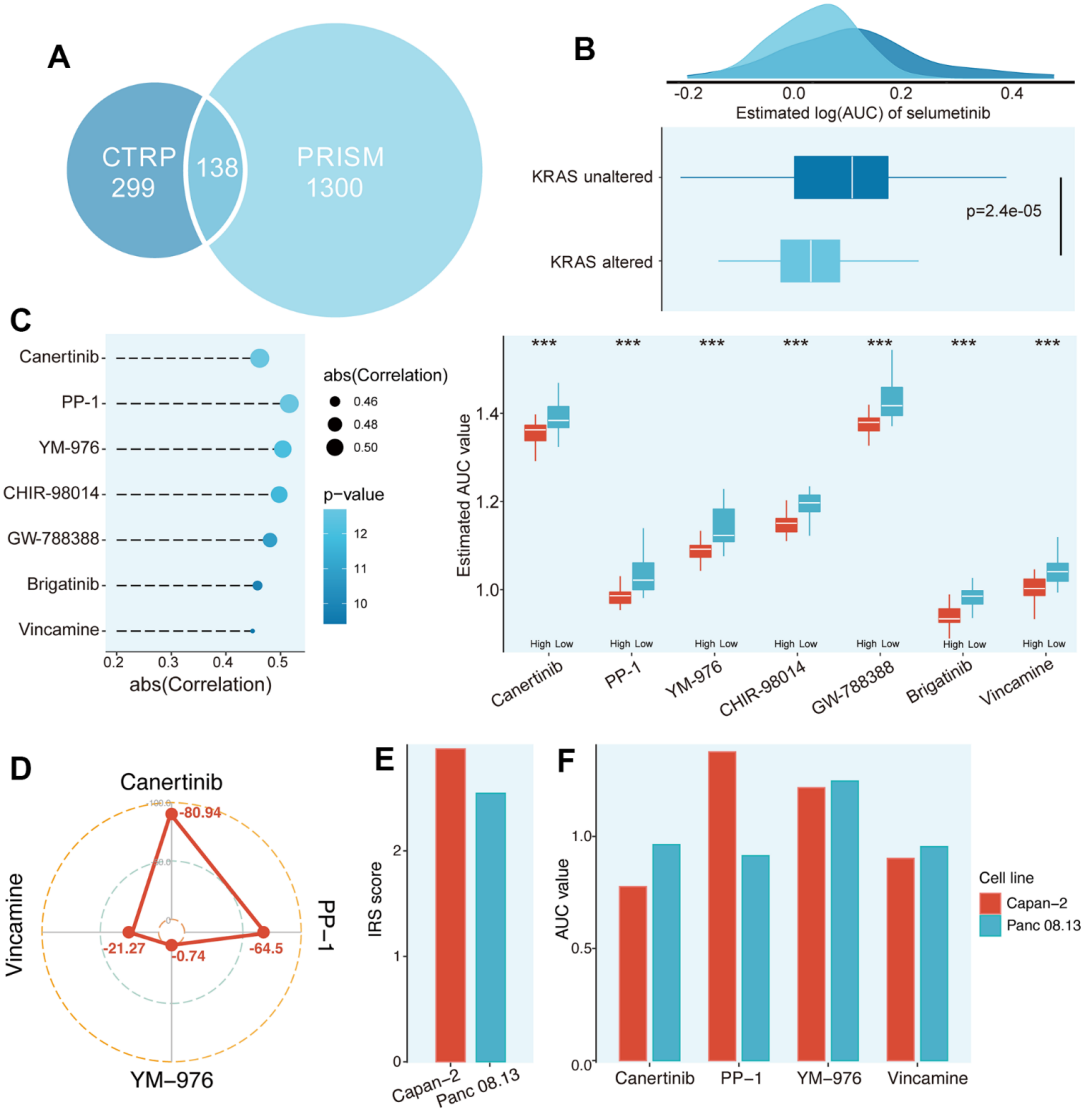
**Identification of potential agents for high IRS score PCs.** (**A**) Data availability of drug sensitive data in CTRP and PRISM datasets. (**B**) Validation of the predicted drug sensitive data based on literature. (**C**) Potential agents for high IRS patients were identified from CTRP and PRISM datasets. (**D**) The CMap score of four candidate compounds. (**E**) The IRS score between Capan-2 and Panc 08.13 cell lines. (**F**) The AUC value of four candidate compounds in Capan-2 and Panc 08.13 cell lines.

In the past decade, high-throughput sequencing analysis of large samples has greatly advanced the molecular biology of PC. Hence, we try to detect the potential small molecular compounds for high IRS PCs. The information on compounds in the Cancer Therapeutics Response Portal (CTRP) and Profiling Relative Inhibition Simultaneously in Mixtures (PRISM) database were selected for subsequent analysis after removing the duplicated compound information in the two databases (excluding hematopoietic and lymphoid tissue-derived CCLs) (Figure 8A).

For drug response prediction, many machine learning (ML) methods have been reported, ranging from multivariate linear regression and support vector machine (SVM) to RF and k-nearest neighbours (KNN). Among ML methods, linear regression methods, such as ridge regression and elastic net, tend to exhibit good and robust performance in different settings [16]. Therefore, ridge regression model located in the “oncoPredict” package, which has been applied to multiple studies and proven to be reliable, was applied to estimate drug response of clinical samples in this study [17]. Before selecting the compounds, we further validated the predicted drug sensitivity (AUC) in our cohort. Selumetinib, a PI3K pathway inhibitor, was reported to improve the prognosis in the treatment of KRAS-mutant patients compared to those without KRAS mutations [18]. Thus, we classified PC patients into KRAS altered and KRAS unaltered subgroups. The AUC of PC patients in the KRAS altered group was significantly decreased (Figure 8B, P = 2.4e-05), which was consistent with the clinical findings of Simertinib above. Finally, 1 compound from the CTRP database (Canertinib) and 6 compounds from the PRISM database (PP-1, YM-976, CHIR-98014, GW-788388, Brigatinib and Vincamine) were obtained following the protocol described in Materials and Methods (Figure 8C).

Although these 7 compounds had lower predictive AUC values in the samples with higher PPS scores and their predictive AUC values were significantly negatively correlated with IRS scores, the above analysis alone could not support the conclusion that these compounds had therapeutic effects on PCs. Hence, CMap analysis was utilized to find the most reliable compounds. Among the 7 candidate compounds identified before, Canertinib and PP-1 showed relatively low CMap scores (Canertinib, -80.94; PP-1, -64.5), indicating its therapeutic potential (Figure 8D and Supplementary Table 7). To further test the efficiency of these candidates, two PC cell lines (Capan-2 and Panc 08.13) in CTRP and PRISM have been extracted for the following analysis. We first calculated the IRS score of these two cell lines, and the Capan-2 possessed a relatively higher IRS score than Panc 08.13 (Figure 8E and Supplementary Table 8). Secondly, the AUC value of these candidates between Capan-2 and Panc 08.13 were compared. The results indicated that only the AUC value of Canertinib was significantly lower in the Capan-2 compared to Panc 08.13, implying that Canertinib might be the promising potential treatment compound targeted high IRS score PCs (Figure 8F and Supplementary Table 8).

## DISCUSSION

As the most common malignant tumor among solid tumors, the complex crosstalk in the microenvironment of pancreatic cancer poses a serious challenge for personalized treatment of patients [19]. With the development of high-throughput sequencing analysis, subtyping cancers on the basis of molecular similarities and clinical characteristics could improve the existing morphological and imaging methods for personalized treatment and risk stratification [20]. Up to now, PC can be divided into multiple molecular subtypes (MS), including Bailey’s classification, Collisson’s classification, Moffitt’s tumor classification, Moffitt’s stromal classification, Puleo’s classification and Li’s classification. Bailey’s classification includes Squamous, Pancreatic progenitor, Immunogenic, and Aberrantly Differentiated Endocrine Exocrine (ADEX). Among them, the Squamous subtype enriched for inflammation, metabolic reprogramming, cell proliferation and epigenetic downregulation of endodermal genes, which possessed the worst prognosis [21]. Collisson’s classification includes Classical subtype related to adhesion and epithelialization, Exocrine-like subtype related to mesenchymal transition, and QM-PDA related to tumor-derived digestive enzymes [22]. Moffitt’s tumor classification includes the Classical subtype and Basal-like subtype, and the latter is associated with poor survival of PC [23]. Moffitt’s stromal classification contains Absent, Activated and Normal subtypes [23]. Puleo’s classification includes Desmoplastic, Immune classical, Pure basal-like, Pure classical and Stroma Activated subtypes [24]. Li’s classification defines the TIME of PC as Immune Class and Nonimmune Class [25]. However, the molecular typing of PC is in its infancy. Hence, novel molecular signatures are still necessary to provide opportunities to advance the therapeutic development of PC.

It is reported that the interactions between cancer cells and proximal immune cells can ultimately lead to an environment that promotes the growth and metastasis of PC [26]. Furthermore, a deeper understanding of IRGs involved in the TIME could help illustrate their regulatory mechanisms in TIME and develop novel treatment strategies. Numerous types of research have demonstrated that immune and stromal cells are two major components of TIME [27–29]. Hence, we identified the TIME-related differentially expressed IRGs particularly from PC samples by evaluating tumor-infiltrating immune cells and stromal cells via the ESTIMATE algorithm. Additionally, identifying IRGs that are differentially expressed in tumor and normal tissues could be conducive to selecting dysregulated genes in PC. Therefore, we extracted IRGs with differential expression in the TIME and PC samples that may effectively reflect the characteristics of TIME in PC. We further confirmed their functions in the immune system via pathway enrichment analysis. Unsupervised consensus clustering algorithm was implemented to classify PC patients into two TIME subtypes. TIMER analysis exhibited that the Immune_rich subtype possessed higher infiltration of CD8+ T cells. Currently, according to the specific tumor environment and immune contexture, three main subtypes of tumor; the immune hot, altered and cold tumors were determined [30]. The terms “hot” and “cold” are defined by T cell-infiltrated, inflamed but non-infiltrated, and non-inflamed tumors [31]. Hence, the Immune_rich subtype was correlated to the “hot” tumor and the TIDE algorithm exhibited that the “hot” tumor tended to gain lower TIDE scores, implying their sensitivity to ICB treatment was higher. Moreover, the immune signatures of T cells (TH1, IFNγ, GNLY, PRF1, GZMs) were associated with prolonged survival and more sensitive to anti-PD1 treatment [32–35]. Although the abundance of T cells was higher in Immune_rich subtype, most of them were in a dysfunctional state, leading to a lower TIDE score. Therefore, the risk stratification merely based on the infiltration of T cells is too limited to guide the clinical strategies of immunotherapy, and our novel classification may pose new directions in the future.

Despite the rapid development of diagnostic methods and therapeutic strategies for PC, the high degree of heterogeneity in PC still makes its prognosis prediction and treatment efficacy face great challenges. In the past decade, many researchers have done a lot of work to develop immune-related prognostic prediction models. However, the construction method of those prognostic markers is relatively single and only applies to the whole PC population, without individualized clinical management analysis for high-risk groups, which is not enough for accurate risk stratification of PC patients. In fact, with the rapid development of artificial intelligence in the biomedical field, machine learning, as an important branch of artificial intelligence, has been widely used in bulk transcriptomics, single-cell transcriptomics, spatial transcriptomics, radionics and other fields. Chen P [36] and his team showed that through deep learning models (belonging to the branch of machine learning), they developed a model named DeepMACT, which can systematically analyze the size, shape, spatial distribution and other characteristics of tumors, as well as the degree of targeted metastasis by therapeutic monoclonal antibodies. It is an important discovery of the target antibody in the preclinical stage. Boris V J et al. [37] summarized the important role of image-based machine learning algorithms in predicting the clinical outcome of PDAC patients. Among 25 studies based on machine learning algorithms published from 2019 to 2020, 9 models effectively predicted the clinical outcome (AUC: 0.78-0.95, C-index: 0.65-0.76). Therefore, in order to develop a quantified signature to stratify PC patients, we selected machine learning algorithms to construct the IRS model to determine immune-related risk classification in PC patients. Among the 8 genes in the IRS model, SYT12 is reported to play a vital role in oral squamous cell carcinoma (OSCC) progression via CAMK2N1 and could be a new target for OSCC patients [38]. TNNT1 is regulated by miR-873 and confirmed as an oncogene of colorectal cancer (CRC) [39]. TRIM46, which is affiliated in the tripartite motif (TRIM) protein family, acts as an E3 ligase that targets HDAC1 and promotes carcinogenesis and chemoresistance in breast cancer [40]. Similarly, an integrative genomic analysis revealed that SMPD3 is a tumor suppressor gene that could influence the aggressiveness of the hepatocellular carcinoma (HCC) [41]. ANLN promotes the progression of PC via EZH2/miR-218-5p/LASP1 axis, suggesting that ANLN could be served as a potential therapeutic target in PC [42]. CXCL9 was listed as a conserved 4-chemokine signature marks resectable and metastatic PC tumors with an active antitumor phenotype [43].

Meanwhile, we also explored the relationships between IRS and TIME. The IRS score was positively correlated with neutrophils, MDSCs and M2 macrophages, while negatively related to CD8+ T cells and CD4+ T cells. Neutrophils, accounting for 70% of circulating leukocytes, exhibit an N1 (tumor-suppressive) or N2 (tumor-promoting) phenotype in the context of cancer [44]. We suggested that N1 type of neutrophils were abundant in high IRS patients according to the results mentioned above. MDSCs could lead to immunosuppression, including T cell suppression and innate immune regulation via multiple mechanisms in TIME [45]. Most importantly, MDSCs strengthened cell stemness and promoted the metastatic process by promoting EMT through IL-6 secretion in tumors [46]. Macrophages can be polarized into inflammatory M1 (classically activated) or immune-suppressive M2 (alternatively activated). Based on the secretion of IL-4, TIME enhanced the immune suppressive M2 which in turn enables tumor growth and progression [47]. CD8+ T cells along with CD4+ T cells are contributed to adaptive immunity and anti-tumor immunity [48]. scRNA-seq was also applied to further explore the underlying mechanism of how IRS leads to the diversity of TIME. The malignant cells tended to possess higher IRS score and may contribute to the specific TIME in IRS. Additionally, Cell-cell communications illustrated that fibroblast and ductal cells could contribute to the development of tumor cells by targeting the SPP1-CD44 and MIF-CD74 axis. In general, our IRS performed well in predicting prognosis and the sensitivity of immunotherapy in PC patients. However, the ultimate goal of clarifying risk stratification is to achieve individualized and personalized treatment, so the screening of drug targets and potential agents has become the main breakthrough.

With the development of next-generation sequencing genomics, researchers can rapidly identify genetic differences between tumor cells and normal cells, genomic mutations, and changes in downstream pathways, which provides convenience for the development of drug targets. Currently, many types of malignant tumors (e.g., breast and ovarian cancer) benefit from “precision medicine” with targeted drugs. However, few targeted drugs have been approved for PC, and it only marginally prolongs patient survival [49]. Hence, based on pharmacogenomic databases, we identified 8 drug targets and 1 potential agents for high IRS patients in PC.

In terms of 8 targets screened from Drug Repurposing Hub and CCLE datasets, it is reported that the high expression of CCNA2 is associated with a worse prognosis in PC and is correlated with advanced tumor stage [50]. Inhibition of EPHB4 combined with radiation can modulate the microenvironment response post-radiation, contributing to increased tumor control in PC [51]. PLOD genes or PLOD family genes also could be served as potential prognostic biomarkers for PC [52]. PLKT1 suppresses PC progression and inhibits NF-κB activity, and targeting PLKT1 can alleviate the sensitivity of immunotherapy in PC [53]. The up-regulation of PANX1 was correlated with poor outcomes and immune infiltration in PC [54]. CCNB1 silencing suppresses cell proliferation and promotes cell senescence by activating the p53 signalling pathway in PC [55]. Although INCENP and NCF2 haven’t been reported in PC, it needs further exploration could concentrate on these two novel targets. Moreover, we identified Canertinib as the most reliable agent targeting high IRS score PCs based on CTRP and PRISM datasets. Canertinib, an EGFR inhibitor, has been demonstrated effective in pNETs according to available genetic atlas data [56]. But unfortunately, its clinical efficiency in PC has been insofar moderate. Current work provides new insights into improving the therapeutic effect of PC, offering new directions for the precision treatment of PC.

Importantly, our study differed from previous studies in the following aspects: (1) Our established TIME subtype tightly correlated with the classical six classifications, which confirmed the reliability of our classification and shed light on a novel strategy for the treatment of PC. (2) Via multiple machine-learning algorithms, the IRS was constructed and achieved better performance in risk stratification than previous prognostic signatures. (3) Recently, numerous studies have merely focused on subtyping PC at an immunogenic level. However, they failed to deliver precision medicine for PC patients based on their classifications. Apart from being informative regarding TIME and prognosis, IRS can also be implemented for precise oncology, and our results have the potential to refine the status quo of population-based therapies and guide personalized treatment in PC. However, this study has several limitations. For instance, our research merely focused on public retrospective datasets, and the predictive efficiency of the IRS in immunotherapy response requires further validation in immunotherapy cohorts of PC. Furthermore, the results of drug targets and agents prediction cannot be verified against each other, which reduces the power of the conclusions.

In conclusion, we classified two TIME subtypes with specific tumor microenvironments and accessed the differences in potential response among these two subtypes. Additionally, we developed a novel immune-related prognostic signature—IRS, and validated it in various cohorts and experiments. Finally, based on multiple drug susceptibility and target databases, we have identified seven potential therapeutic targets and two compounds, which shed new light on the application of precision medicine in PC.

## MATERIALS AND METHODS

### Data acquisition and preprocessing

The expression profile of pancreatic cancer patients was downloaded from TCGA dataset (https://portal.gdc.cancer.gov/) in the form of Fragments Per Kilobase Million (FPKM) and transformed into log2(TPM+1) format data. The corresponding clinical data from the TCGA dataset was downloaded from UCSC Xena (https://xenabrowser.net/datapages/). A total of 149 cases in TCGA with corresponding PC tissues and complete clinical data were enrolled in the study [57]. The RNA-Seq data of the CELL cohort (CPTAC3-Discovery project, n = 135) was employed in this study to construct the prognostic signature, which was obtained from Proteomic Data Commons (PDC, https://pdc.cancer.gov/pdc/) and LinkedOmics (http://www.linkedomics.org/data_download/CPTAC-PDAC/). TCGA and CELL cohorts were combined as a meta-cohort (n = 284) to facilitate the model training and the “sva” R package was used to remove the batch effect between two independent datasets (Supplementary Figure 5). Genotype-Tissue Expression (GTEx, https://www.gtexportal.org/) dataset containing the expression data of normal pancreatic was also included (n = 167). Meanwhile, ICGC (https://dcc.icgc.org/, n = 81) and GEO (http://www.ncbi.nlm.nih.gov/geo) datasets (GSE62452, https://www.ncbi.nlm.nih.gov/geo/query/acc.cgi?acc=GSE62452, n = 65) were extracted to validate the efficiency of an established model. The single-cell dataset of PDAC (CRA001160) was extracted from the TISCH database (http://tisch.comp-genomics.org/home/), which contained 24 pancreatic tumor tissues and 11 normal tissues. In terms of studies in personalized treatment, the expression profile of pancreatic CCLs was screened from the Broad Institute CCLE project (https://portals.broadinstitute.org/ccle/, n = 44). Drug sensitivity data of CCLs were achieved from the CTRP v.2.0 (https://portals.broadinstitute.org/ctrp), containing the sensitivity data for 481 compounds over 835 CCLs) and PRISM Repurposing dataset (19Q4, released December 2019, https://depmap.org/portal/prism/, containing the sensitivity data for 1448 compounds over 482 CCLs).

### Screening for immune-related genes

ESTIMATE algorithm was applied to calculate the immune scores and stromal scores based on the expression profile of meta-cohort [58]. Then, according to the median value, PC patients were divided into high- and low-immune/stromal score subgroups. Differential expression analysis was performed to screen out the dysregulatory genes among immune score subgroup, stromal score subgroup, and between tumor and normal pancreatic samples via “DESeq2” R package with the criteria of |log2Fold change| >1 and p<0.05 [59]. Importantly, the converged DEGs among three subgroups were defined as IRGs.

### Unsupervised clustering analysis

In order to determine the specific patterns of IRGs, the unsupervised consensus clustering algorithm was implemented via “ConsensusClusterPlus” R package [60]. In addition, principal component analysis (PCA) analysis was also conducted to validate the difference between subtypes.

### Enrichment analysis and immune landscape of immune subtypes

GSEA, GO and KEGG annotation were adopted for determining the statistical significance of molecular pathways as well as the consistent heterogeneities between among different groups via “clusterProfiler” R package [61]. A pathway with FDR q < 0.25 and P < 0.05 was defined as statistically significant. single sample Gene Set Enrichment Analysis (ssGSEA) [62] and TIMER [63] algorithm was utilized to evaluate the tumor-infiltrating immune cells among different TIME subtypes. The previously published signatures of immune- and stroma- cells were selected to calculate the abundance of tumor-infiltrating immune cells through ssGSEA [62]. TIDE algorithm was used to predict responsiveness to ICBs between different groups, and lower TIDE scores implied better immunotherapeutic efficacy [64].

### Published PC classifications prediction and comparison

The relationship between our TIME subtype and reported PC molecular classifications was also explored. Six classical PC classifications have been analyzed, including Bailey’s classification [21], Collisson’s classification [22], Moffitt’s tumor classification [23], Moffitt’s stromal classification [23], Puleo’s classification [24] and Li’s classification [25]. Based on published signature genes and algorithms, unsupervised consensus clustering was applied for identifying the subtyping schemas of Bailey’s classification, Collisson’s classification, Moffitt’s tumor classification, Moffitt’s stromal classification and Li’s classification on meta-cohort using the “ConsensusClusterPlus” package in R. For the prediction of Puleo’s classification, we followed the pipeline defined by Puleo [24]. For each sample in meta-cohort, the expression of genes in the centroids was selected, and Spearman rank correlation analysis was conducted between selected genes and 5 centroids. The subtype centroid with the highest correlation is the predicted class of the tested sample. The comparison between the distribution of six predicted classifications and our TIME subtype was measured by Fisher’s exact test. R package “ggalluvial” was utilized to plot the Sankey diagram [65]. Cramer’s V served as an effect size measurement for the association between TIME subtypes and the other six classifications. It ranges from 0 to 1 where, 0 indicates no association between the two variables, and 1 indicates a perfect association between them.

### Screening, construction and validation of the IRS

PC patients in meta-cohort were categorized into the training set and testing set at the ratio of 7:3. Then, in order to screen the prognostic DEGs, the Bootstrapping univariate Cox analysis was conducted by the “survival” R package. Furthermore, RSF analysis was applied to dimension reduction. The highest C-index of out-of-bag samples was used as the best model and the underlying gene set was observed, and this gene set was defined as IRS. Finally, The IRS scoring model was constructed with the correlation coefficients obtained from multivariate Cox regression, and the formula was as follows:


$$IRS\;score=\sum_{i=1}^{n}Coef_{i}*x_{i}$$

Where Coef_i_ is the multivariate Cox regression coefficient, and x_i_ is the expression value according to the optimal IRS score, patients were divided into IRS_high and IRS_low group. The area under the curve (AUC) value was used as the criteria to evaluate the effectiveness of the IRS model.

Otherwise, three conventional published signatures (Wang’s signature [66], Tao’s signature [67] and Dai’s signature [68]) and two classical prognostic signatures (PAMG [69] and PurIST [70] signature) in PC were collected to compare the predictive accuracy of the IRS and these signatures. For three conventional signatures, we calculated the risk scores based on the genes and coefficients provided by the articles (Supplementary Table 9). The PAMG score was calculated via the “pdacmolgrad” R package. PurIST score and classification were obtained following the protocol in the original publication [70]. Afterwards, we comprehensively assessed their predictive performance based on AUC values.

### IRS-based chemotherapy sensitivity and ICB sensitivity analysis

To predict potential therapeutic effects in different subgroups, “oncoPredict” R package [17] was applied to predict the drug response of PC patients. Moreover, several predicted scores were conducted to evaluate the immunotherapeutic response to IRS, namely, ICB expression, TMB, TME score and IPS. TMB score was computed based on the somatic mutation data from the TCGA dataset, and confirmed as a predictor of immunotherapy. IPS score was downloaded from the cancer immunome group atlas (TCIA, https://tcia.at/home) after uploading the expression profile of patients [62]. TME score was calculated by “TMEscore” R package [71], revealing the patients’ response to ICB. To further validate the role of IRS in the prediction of immunotherapy, we implemented the Subclass mapping (SubMap) to evaluate the expression similarity between IRS_high/IRS_low patients and patients who responded/non-responded to anti-PD-1 and anti-CTLA4 immunotherapy [72].

### Single-cell RNA sequencing analysis

The dataset CRA001160 was utilized for scRNA sequencing analysis. UMI count matrices were generated for each sample, and imported into the “Seurat” R package. Low quality cells (<200 genes/cell or >20% mitochondrial genes) were excluded. “Seurat” package was applied for normalization and scaling of the expression matrix, using default settings [73]. Mitochondrial contamination was regressed out by setting “vars.to.regress” parameter. The doublets were cleared out by the “DoubletFinder” R package (version 2.0.3) [74]. To reduce the dimensionality of the expression matrix, PCA analysis was performed based on 2,000 highly variable genes. JackStraw analysis was utilized to identify significant principal components (PC), and PC 1~10 was used for graph-based clustering (res = 0.8) to determine distinct groups of cells. Via previously computed PC 1~10, these groups were projected onto the t-SNE analysis. Subsequently, we used the Seurat FindMarker function to find marker genes of each cell cluster, and defined cell types based on previous datasets and literature [75–78].

### Cell–cell interaction analysis

To analyze the cell-cell interactions, R package “CellChat” [79] was employed to predict the major incoming and outgoing intercellular communication networks. In our work, cell-cell interactions were analyzed following the default pipeline. Normalized scRNA-seq counts data were used to create CellChat object with the recommended preprocessing functions. CellChatDB.human was utilized as the database for inferring cell–cell communication with default parameters. “ECM-Receptor” in the database was applied in the analysis. Communications including less than 10 cells were excluded. “iTALK” R package [80] was also used to estimate cell-cell communication. The top 50% of highly expressed genes in each cluster were projected to ligand-receptor pairs in the “iTALK” package. Four categories, including checkpoint protein, cytokine, growth factor, and “other” protein, were employed in our study. The top 30 ligand-receptor pairs for each type were extracted for visualization.

### Exploring the biological process of IRS from gene-level to pathway-level

Pathifier analysis was implemented to dig into the differences between IRS_high and _low subgroups via “Pathifier” R package [81]. The Pathifier analysis method is used to identify specific signaling pathways at specific stages of cancer, and can be used in the personalized treatment of cancer. By means of correlation, variance stability and principal component analysis, Pathway Deregulation Score (PDS) was calculated for each PC sample, and then used to estimate the degree to which the activity of a pathway in PC samples deviates from normal samples.

### RT-qPCR analysis

All cells in the experiments, including AsPC-1 (RRID: CVCL_0152), BxPC-3 (RRID: CVCL_0186), PANC-1 (RRID: CVCL_0480), PaTu 8988t (RRID: CVCL_1847), and hTERT-HPNE (RRID: CVCL_C466) cells, were purchased from the Cell Bank of the Chinese Academy of Sciences (Shanghai, China) and used for RT-qPCR. All human cell lines have been authenticated using STR profiling within the last three years and that all experiments were performed with mycoplasma-free cells. RNA was reverse transcribed into cDNA using a reverse transcription kit. Gently vortex and then put into the quantitative PCR instrument for amplification. Three technical replicates of each PCR reaction were conducted to ensure the credibility of the experiment. The forward and reverse primers were listed in Supplementary Table 10.

### Statistical analysis

Student’s t-test was applied in the normal distribution data; Wilcox test was applied for non-normal distribution data between independent groups. Spearman analysis was applied to estimate the correlations between two variables that are not linearly related. The Kaplan–Meier test was utilized to validate the fraction of PC patients living for a certain survival time via the survival package. The log-rank test was conducted to compare the significance of the difference. A two-tailed p-value of less than 0.05 was deemed statistically significant unless specifically stated. See Supplementary File 1 for more information.

## Supplementary Material

Supplementary Figures

Supplementary Table 1

Supplementary Table 2

Supplementary Table 3

Supplementary Table 4

Supplementary Table 5

Supplementary Tables 6-10

Supplementary File 1
