# Supplementary material for: Machine learning algorithm integrates bulk and single-cell transcriptome sequencing to reveal immune-related personalized therapy prediction features for pancreatic cancer
Source: Aging (Albany NY). 2023 Dec 12;15(23):14109–40. doi: 10.18632/aging.205293 (PMC10756117; doi:10.18632/aging.205293)
Supplement: Supplementary Figures [file aging-15-205293-s001.pdf]

SUPPLEMENTARY FIGURES

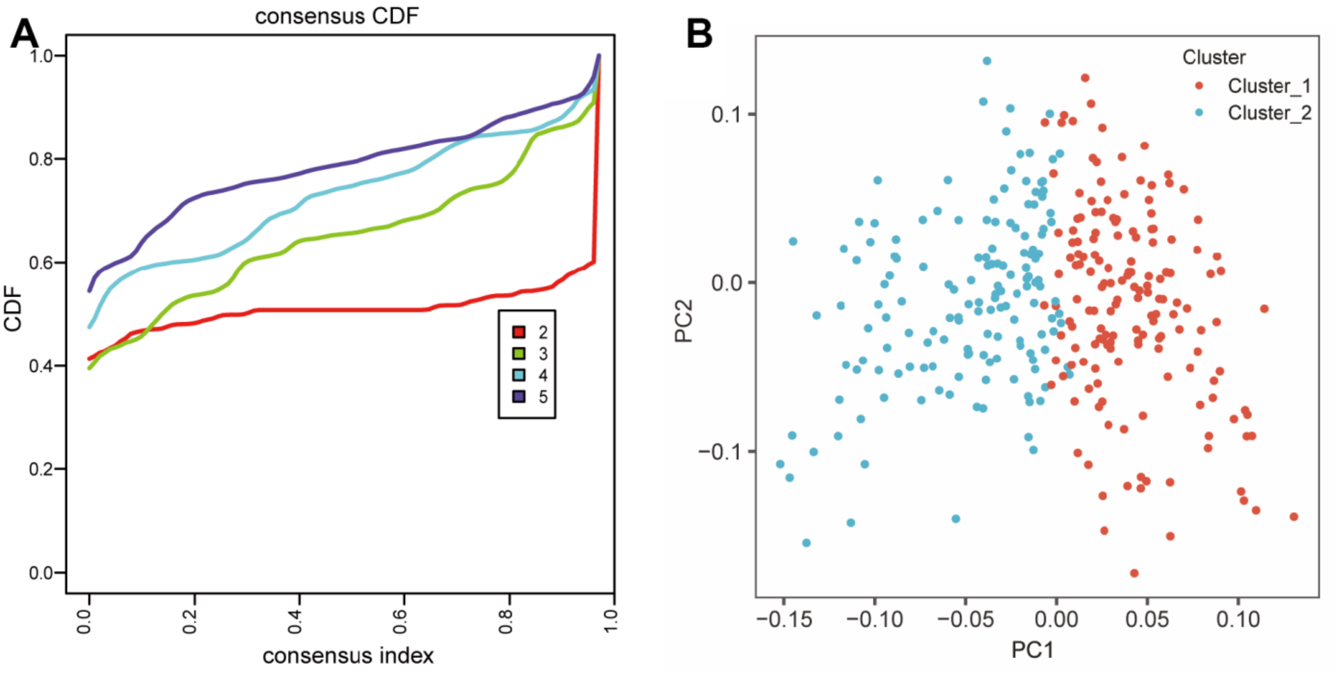

Supplementary Figure 1. Identification of TIME-related subtypes. (**A**) The CDF plot of consensus clustering. (**B**) The PCA plot demonstrated the differences between the two clusters.

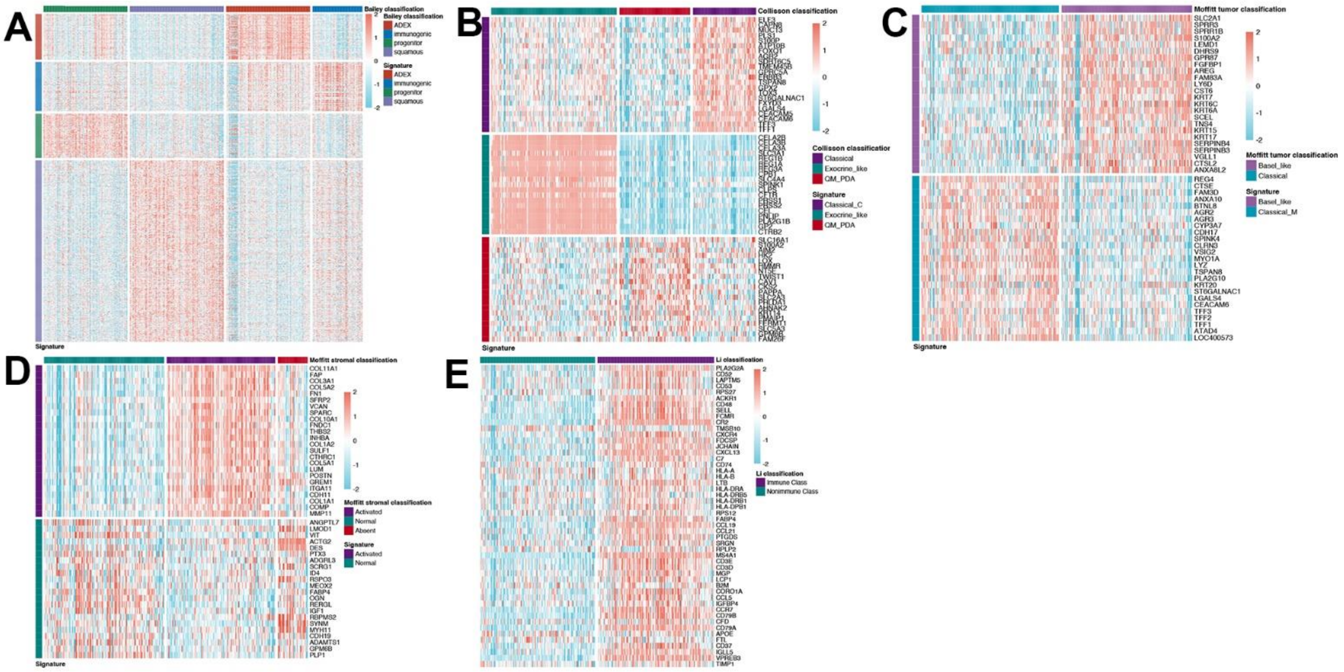

Supplementary Figure 2. Published PC subtypes prediction. Heatmap showed that we defined Bailey's classification (**A**), Collisson's classification (**B**), Moffitt's tumor classification (**C**), Moffitt's stromal classification (**D**) and Li's classification (**E**) based on the published classifier exemplar genes.

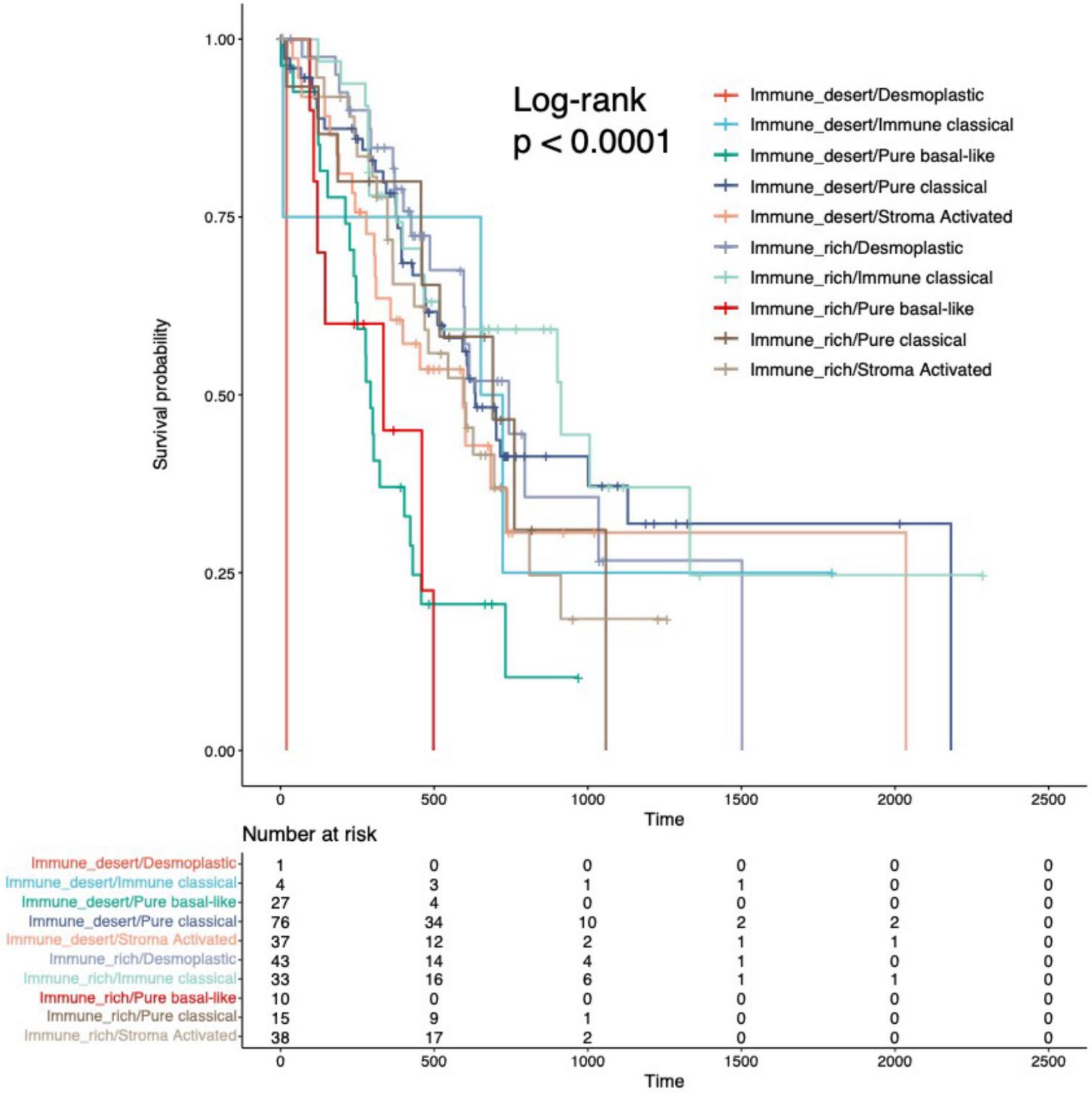

Supplementary Figure 3. Kaplan-Meier curves of overall survival were plotted according to the TIME subtype and Puleo's classification.

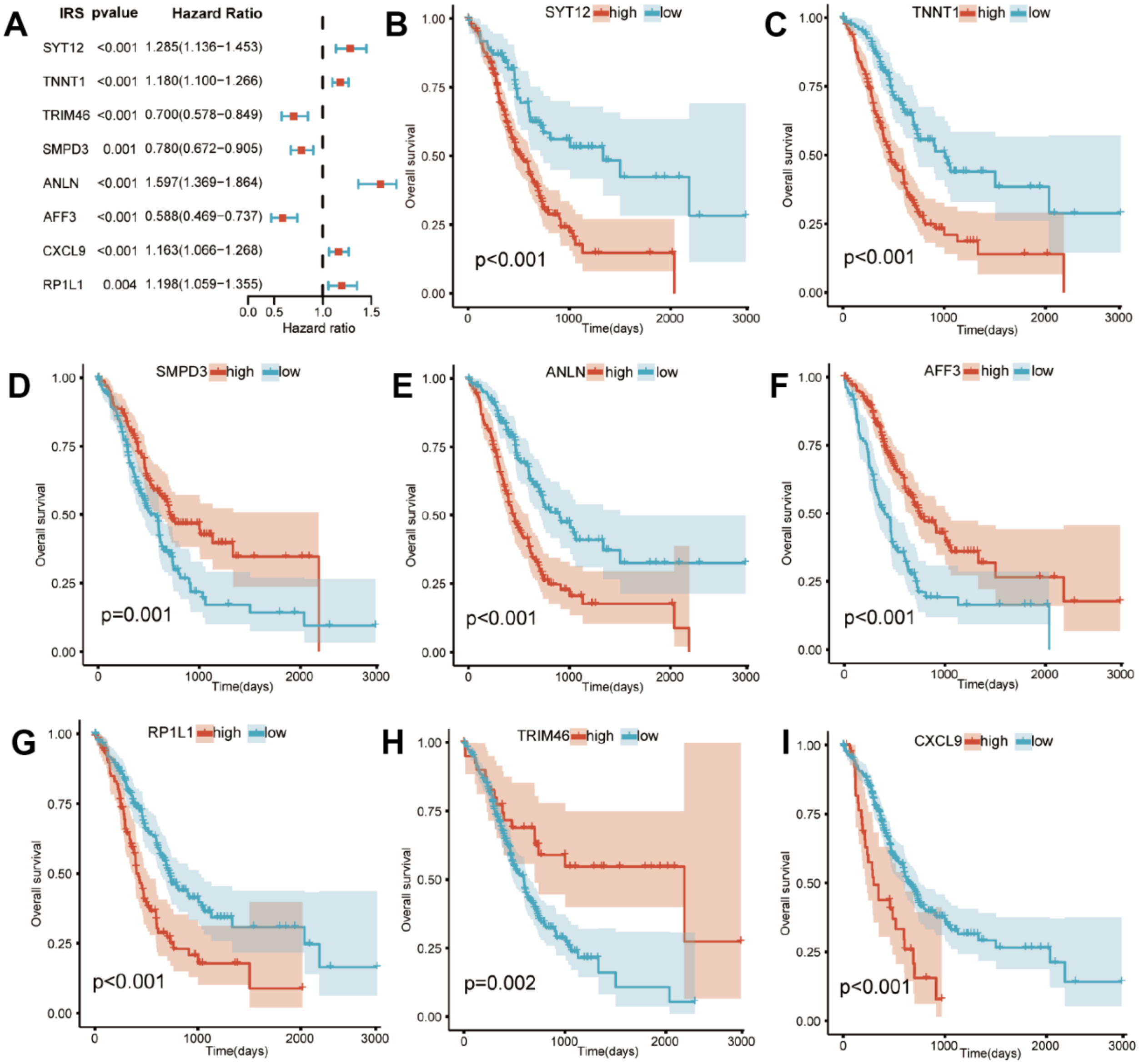

Supplementary Figure 4. Validation of IRS. (**A**) Forest plot of univariate cox analysis based on the training set. (**B**–**I**) The survival analysis is based on the expression of IRS genes.

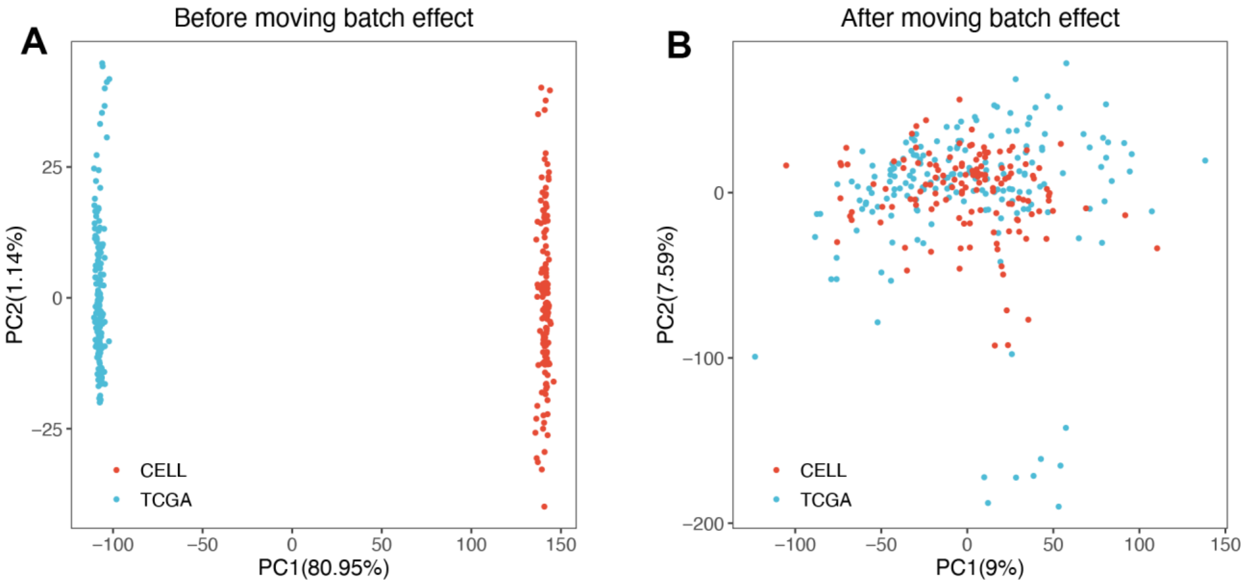

Supplementary Figure 5. Evaluation of the results of batch effect correction. The principal component analysis (PCA) before (**A**) and after (**B**) batch effect correction on Meta-cohort.
