# Supplementary material for: Machine learning algorithm integrates bulk and single-cell transcriptome sequencing to reveal immune-related personalized therapy prediction features for pancreatic cancer
Source: Aging (Albany NY). 2023 Dec 12;15(23):14109–40. doi: 10.18632/aging.205293 (PMC10756117; doi:10.18632/aging.205293)
Supplement: Supplementary Tables 6-10 [file aging-15-205293-s007.pdf]

Supplementary Table 6. Comparison of the TIME subtype with other pancreatic molecular classifications.

Classifications		Overall	Immune_desert	Immune_rich	P-value
		284	145	139	
**TIME subtype (%)**	Immune_desert	145 (51.06)	145 (100.00)	0 (0.00)	<0.0001
	Immune_rich	139 (48.94)	0 (0.00)	139 (100.00)	
**Bailey's classification (%)**	ADEX	85 (29.93)	55 (37.93)	30 (21.58)	<0.0001
	Immunogenic	54 (19.01)	5 (3.45)	49 (35.25)	
	Progenitor	61 (21.48)	59 (40.69)	2 (1.44)	
	Squamous	84 (29.58)	26 (17.93)	58 (41.73)	
**Collisson's classification (%)**	Classical	69 (24.30)	49 (33.79)	20 (14.39)	0.0006
	Exocrine_like	138 (48.59)	60 (41.38)	78 (56.12)	
	QM_PDA	77 (27.11)	36 (24.83)	41 (29.50)	
**Moffitt's tumor classification (%)**	Basel_like	133 (46.83)	70 (48.28)	63 (45.32)	0.7044
	Classical	151 (53.17)	75 (51.72)	76 (54.68)	
**Moffitt's stromal classification (%)**	Absent	46 (16.20)	30 (20.69)	16 (11.51)	0.0101
	Activated	115 (40.49)	64 (44.14)	51 (36.69)	
	Normal	123 (43.31)	51 (35.17)	72 (51.80)	
**Puleo's classification (%)**	Desmoplastic	44 (15.49)	1 (0.69)	43 (30.94)	<0.0001
	Immune classical	37 (13.03)	4 (2.76)	33 (23.74)	
	Pure basal-like	37 (13.03)	27 (18.62)	10 (7.19)	
	Pure classical	91 (32.04)	76 (52.41)	15 (10.79)	
	Stroma Activated	75 (26.41)	37 (25.52)	38 (27.34)	
**Li's classification (%)**	Immune Class	143 (50.35)	48 (33.10)	95 (68.35)	<0.0001
	Nonimmune Class	141 (49.65)	97 (66.90)	44 (31.65)	

Supplementary Table 7. CMap score of candidate potential agents.

Compounds	CMap score
Canertinib	-80.94
PP-1	-64.5
YM-976	-0.74
CHIR-98014	NA
GW-788388	NA
Brigatinib	NA
Vincamine	-21.27

Supplementary Table 8. AUC values of two PC cell lines.

DepMap_ID	Cell line	RRID	IRS score	Group	Canertinib	PP-1	YM-976	Vincamine
ACH-000417	Capan-2	CVCL_0026	2.96757	IRS_high	0.77602	1.3768761	1.216856	0.8804061
ACH-000107	Panc 08.13	CVCL_1638	2.546593	IRS_low	0.96352	0.9137654	1.246992	0.9541501

Supplementary Table 9. Details of 3 published signatures in PC.

Model	PMID	Type	Author	Coef	Gene
Model-1	35953822	mRNA	Wang C	0.0893	ALB
Model-1	35953822	mRNA	Wang C	0.1935	CXCL10
Model-1	35953822	mRNA	Wang C	0.0635	IAPP
Model-1	35953822	mRNA	Wang C	-0.2633	LIFR
Model-1	35953822	mRNA	Wang C	-0.2338	LYZ
Model-1	35953822	mRNA	Wang C	0.2893	MET
Model-1	35953822	mRNA	Wang C	0.2177	S100A14
Model-2	36118860	mRNA	Tao S	0.2302	GSDMC
Model-2	36118860	mRNA	Tao S	-0.4664	ELANE
Model-2	36118860	mRNA	Tao S	0.3341	IL18
Model-2	36118860	mRNA	Tao S	-0.4324	NLRP1
Model-2	36118860	mRNA	Tao S	0.1297	NLRP2
Model-3	35831362	mRNA	Dai L	0.132	S100P
Model-3	35831362	mRNA	Dai L	0.098	S100A2
Model-3	35831362	mRNA	Dai L	0.095	MMP12

Supplementary Table 10. The forward and reverse primers of hub genes.

Name	Primer(5'to3')
Homo-SYT12-F	CAGAATACCATCTGAGCGTCATC
Homo-SYT12-R	TAGTCGTAATTGGGGAACGGA
Homo-TNNT1-F	TGATCCCGCCAAAGATCCC
Homo-TNNT1-R	TCTTCCGCTGCTCGAAATGTA
Homo-TRIM46-F	TTCCGACCCAAGGGCCTTAT
Homo-TRIM46-R	AGAGTTGACATACCAGGCGTT
Homo-SMPD3-F	GCTGCCCTTTGCGTTTCTC
Homo-SMPD3-R	TCCAGCCGTGAATAGATGTAGG
Homo-ANLN-F	TGCCAGGCGAGAGAATCTTC
Homo-ANLN-R	CGCTTAGCATGAGTCATAGACCT
Homo-AFF3-F	GCCCTACAAGACTAACAAGGGG
Homo-AFF3-R	ACTCCAACGAGATGACTCTGAT
Homo-CXCL9-F	CCAGTAGTGAGAAAGGGTCGC
Homo-CXCL9-R	AGGGCTTGGGGCAAATTGTT
Homo-RP1L1-F	TCCCGGAAGAGTCTTAAAACCC
Homo-RP1L1-R	GCGGCCAGGTTCCTAGTATTC
