# Supplementary material for: Machine learning algorithm integrates bulk and single-cell transcriptome sequencing to reveal immune-related personalized therapy prediction features for pancreatic cancer
Source: Aging (Albany NY). 2023 Dec 12;15(23):14109–40. doi: 10.18632/aging.205293 (PMC10756117; doi:10.18632/aging.205293)
Supplement: Supplementary File 1 [file aging-15-205293-s008.pdf]

Supplementary materials

Identification of potential drug targets

The detailed target information of 6125 compounds was downloaded from The Drug Repurposing Hub database (https://clue.io/repurposing)[1]. After removing the duplicated target information, we conducted the following procedures: 1) we performed the Spearman correlation analysis between the protein expression profile of target genes and IRS score in PC patients, and candidate targets were screened out with the threshold of $R > 0.3$ & $p < 0.05$; 2) we computed the IRS scores of PC cell lines in CCLE dataset ($n = 44$), and Spearman correlation analysis was performed between CERES scores obtained from DepMap database (https://depmap.org/portal/) and IRS scores. CERES is an algorithm to evaluate gene dependency from essentiality screens while correcting the copy-number effect[2]. A lower CERES score of a gene indicates stronger dependence of the gene on the malignant biological phenotype of PC, so $R < -0.3$ and $p < 0.05$ was used as the criterion to screen the drug targets related to the poor prognosis of PC; 3) after intersecting the candidate drug target selected in 1) and 2), the drug targets with high confidence were finally obtained.

Evaluation of drug sensitivity on cell lines

The CTRP and PRISM datasets contain the gene expression profiles and drug sensitivity profiles of hundreds of CCLs, which can be utilized to predict drug sensitivity. Cell lines with a missing value (NA value) of more than 20% or from hematopoietic and lymphoid tissues were excluded, and the "oncoPredict" R package [3]was used to predict the drug sensitivity of PC samples. The following analyses were performed using CTRP and PRISM datasets. We applied the Wilcoxon rank-sum test between the high IRS score subgroup (top 20%) and the low PPS score subgroup (bottom 20%). The compounds with higher sensitivity (lower AUC values) in the subgroup with higher IRS scores (log2FoldChange > 0.03) were identified. Subsequently, Spearman correlation analysis was performed between drug susceptibility values and IRS scores to identify compounds with negative correlation coefficients ($R < -0.45$). In addition, we used the CMap (Connectivity Map, https://clue.io/query) database to explore the compounds targeting the genes associated with the IRS_high subtype[4]. We queried the CMap database and selected the compound with a negative enrichment score and $p < 0.05$. Eventually, the efficiency of these candidates was evaluated in two PC cell lines. Importantly, the compound overlapping in the results of the above analyses was considered a potential treatment for a certain subtype.

Reference:

1. Corsello SM, Bittker JA, Liu Z, Gould J, McCarren P, Hirschman JE, et al. The Drug Repurposing Hub: a next-generation drug library and information resource. Nat Med. 2017;23(4):405-8.

2. Meyers RM, Bryan JG, McFarland JM, Weir BA, Sizemore AE, Xu H, et al. Computational correction of copy number effect improves specificity of CRISPR-Cas9 essentiality screens in cancer cells. Nat Genet. 2017;49(12):1779-84.
3. Maeser D, Gruener RF, Huang RS. oncoPredict: an R package for predicting in vivo or cancer patient drug response and biomarkers from cell line screening data. Brief Bioinform. 2021;22(6).
4. Musa A, Ghoraie LS, Zhang SD, Glazko G, Yli-Harja O, Dehmer M, et al. A review of connectivity map and computational approaches in pharmacogenomics. Brief Bioinform. 2018;19(3):506-23.
